# Plastid establishment did not require a chlamydial partner

**DOI:** 10.1038/ncomms7421

**Published:** 2015-03-11

**Authors:** Daryl Domman, Matthias Horn, T. Martin Embley, Tom A. Williams

**Affiliations:** 1 Department of Microbiology and Ecosystem Science, University of Vienna, A-1090 Vienna, Austria; 2 Institute for Cell and Molecular Biosciences, Newcastle University, Newcastle upon Tyne NE2 4HH, UK

## Abstract

Primary plastids descend from the cyanobacterial endosymbiont of an ancient eukaryotic host, but the initial selective drivers that stabilized the association between these two cells are still unclear. One hypothesis that has achieved recent prominence suggests that the first role of the cyanobiont was in energy provision for a host cell whose reserves were being depleted by an intracellular chlamydial pathogen. A pivotal claim is that it was chlamydial proteins themselves that converted otherwise unusable cyanobacterial metabolites into host energy stores. We test this hypothesis by investigating the origins of the key enzymes using sophisticated phylogenetics. Here we show a mosaic origin for the relevant pathway combining genes with host, cyanobacterial or bacterial ancestry, but we detect no strong case for *Chlamydiae* to host transfer under the best-fitting models. Our conclusion is that there is no compelling evidence from gene trees that *Chlamydiae* played any role in establishing the primary plastid endosymbiosis.

## Abstract (web summary)

Primary plastids descend from an endosymbiosis involving cyanobacteria, an ancient eukaryotic host and, possibly, a chlamydial pathogen. Here, Domman and colleagues use sophisticated phylogenetic methods to show that *Chlamydiae* did not play a role in establishing the primary plastid endosymbiosis.

## Graphical abstract

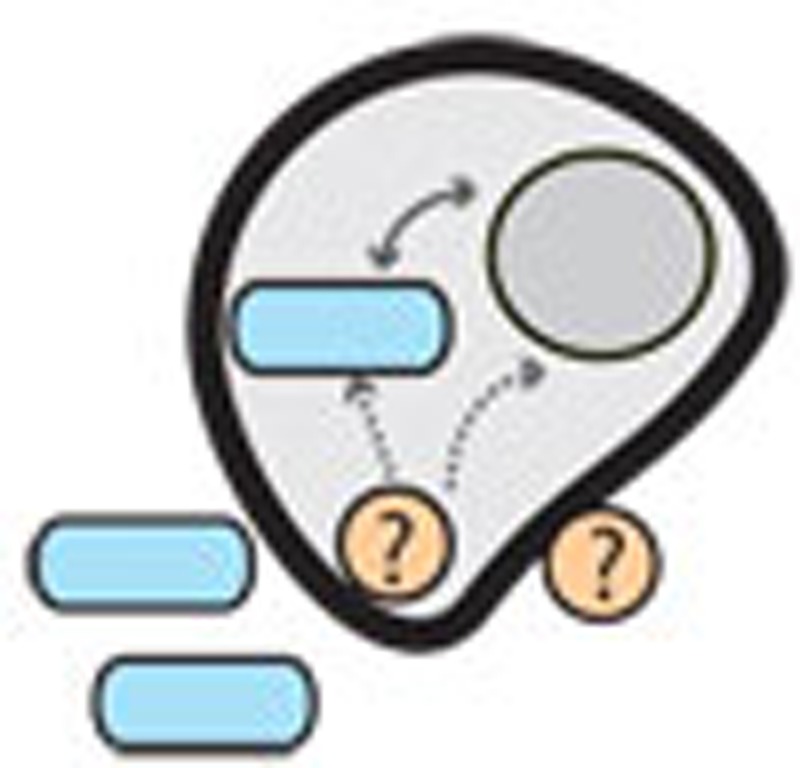

Endosymbiosis is key to the evolutionary success of eukaryotes^1^, from the ancient endosymbiotic origins of mitochondria and chloroplasts^2^ to the methanogenic symbionts of anaerobic ciliates^3^ and the nutritional symbioses of sap-feeding insects^4^. Cell biology and phylogenetics testify to the prokaryotic origins of these endosymbiotic organelles, but the molecular mechanisms by which their free-living progenitors were originally recruited and integrated with a host cell remain poorly understood. The endosymbiotic capture of a cyanobacterium by a heterotrophic eukaryotic host cell at the origin of the Archaeplastida marked one of the most important events in evolutionary history, for through this symbiosis all plant life would emerge. Other photosynthetic eukaryotes obtained their plastids through secondary endosymbiosis of one of these primary lineages, implying that—with a single exception^5^—all photosynthetic eukaryotes trace the origin of their photosynthetic machinery to the primary cyanobacterial endosymbiosis^6^. However, despite substantial progress on the evolution of plastids and their relationships to free-living cyanobacteria^7^,^8^, the initial selective pressure that drove the acquisition and retention of the cyanobacterial endosymbiont remains unclear. Modern plastid and host metabolisms are intimately intertwined, with the chloroplast providing primarily fixed carbon to the host in exchange for a multitude of metabolites, including phosphate derivatives and NAD^9^. However, present-day host–plastid interactions are the product of more than a billion years of co-evolution and the situation may have been very different at the time of the initial endosymbiosis. In addition to the provision of carbohydrates to the host^10^, nitrogen fixation^8^ and the production of molecular oxygen for use by host mitochondria^11^ have also been proposed as initial selective drivers for the retention of the cyanobacterial endosymbiont.

Recently, a detailed, metabolically explicit hypothesis for the initial selective pressure driving endosymbiosis was proposed in which the heterotrophic host cell that engulfed the cyanobacterial endosymbiont was already infected with an ancient member of the *Chlamydiae*^12^,^13^,^14^,^15^,^16^. In this ‘ménage à trois’^16^ (Fig. 1), named with reference to the proposed tripartite nature of the endosymbiosis, the chlamydial partner secreted a series of effectors that manipulated the host cell, rerouting host energy through glycogen metabolism for subsequent conversion to maltotetraose and import into the pathogen^16^. The proposed first step in this process was the conversion of host glucose-1-phosphate to the bacterial metabolite adenine diphosphoglucose (ADP-glucose) by the chlamydial effector GlgC; ADP-glucose was subsequently polymerized to glycogen and then processed for import by the pathogen through a series of downstream reactions catalysed by the effectors GlgA, GlgP and GlgX, all secreted by the pathogen into the host cytoplasm. In this scenario, an engulfed cyanobacterium could have provided immediate relief to the infected host cell through the provision of ADP-glucose generated as a byproduct of its own metabolism, preventing further depletion of host energy stores, that is, the energy sink represented by the chlamydial pathogen would provide the initial selective pressure for capture and retention of the cyanobiont. Although the immediate effect would have been to rescue the host cell, this tripartite metabolic interaction might also have potentiated the development of long-term endosymbiosis by establishing an initial metabolic link between host and cyanobiont, through the incorporation of cyanobacterial ADP-glucose into host glycogen stores.

The ménage à trois idea is a useful hypothesis, because it makes explicit cell biological and phylogenetic predictions that can be tested against currently available data. Modern *Chlamydiae* have a broad host range, from humans (where *Chlamydia trachomatis* is a major cause of sexually transmitted disease) to cattle, fish, isopods and protists^17^. However, extant *Chlamydiae* are not known to infect any members of the Archaeplastida, although the situation may have been different in the distant past^18^. The ‘smoking gun’ for the mitochondrial and plastid endosymbioses was the detection of an organelle^19^, and although there is currently no evidence for a chlamydia-derived organelle in modern Archaeplastida, the chlamydial partner might have been lost from the consortium following horizontal transfer (HGT) of the key metabolic genes to the host nucleus^13^,^14^,^16^. In support of the hypothesis, some modern pathogenic *Chlamydiae* appear to manipulate host metabolism by the secretion of glycolytic enzymes^16^,^20^ and some of the homologues of these enzymes from environmental *Chlamydiae* were shown to be secreted by the type III secretion system in a *Shigella* assay^16^. Further, published gene trees for some archaeplastidal enzymes involved in carbohydrate metabolism show the archaeplastidal sequences emerging from within, or clustering with, the *Chlamydiae*. These include the genes encoding the putative effectors GlgX and GlgA mentioned above. These trees are compatible with a chlamydial origin for key components of plant carbohydrate metabolism, providing phylogenetic support for the ménage à trois hypothesis.

However, the deep internal branches of single gene trees are notoriously difficult to reconstruct, because they are often highly sensitive to the methods used, particularly when inferring phylogenies from anciently diverged sequences^21^,^22^. Standard phylogenetic models make simplifying assumptions about the evolutionary process that are often not met, with potential consequences for the relationships inferred. Here we re-evaluate the phylogenetic evidence for the ménage à trois hypothesis using a range of more complex evolutionary models that incorporate additional features of the sequence data shown to be important by statistical tests of model fit. Analyses using the best-fitting phylogenetic models reveal a mosaic origin for archaeplastidal storage polysaccharide metabolism, raise the possibility that some previous analyses have been misled by simple evolutionary models and suggest that there is no need to invoke a chlamydial contribution to the plastid endosymbiosis.

## Results and Discussion

### Simple methods do not adequately model sequence evolution

**Figure 2:**
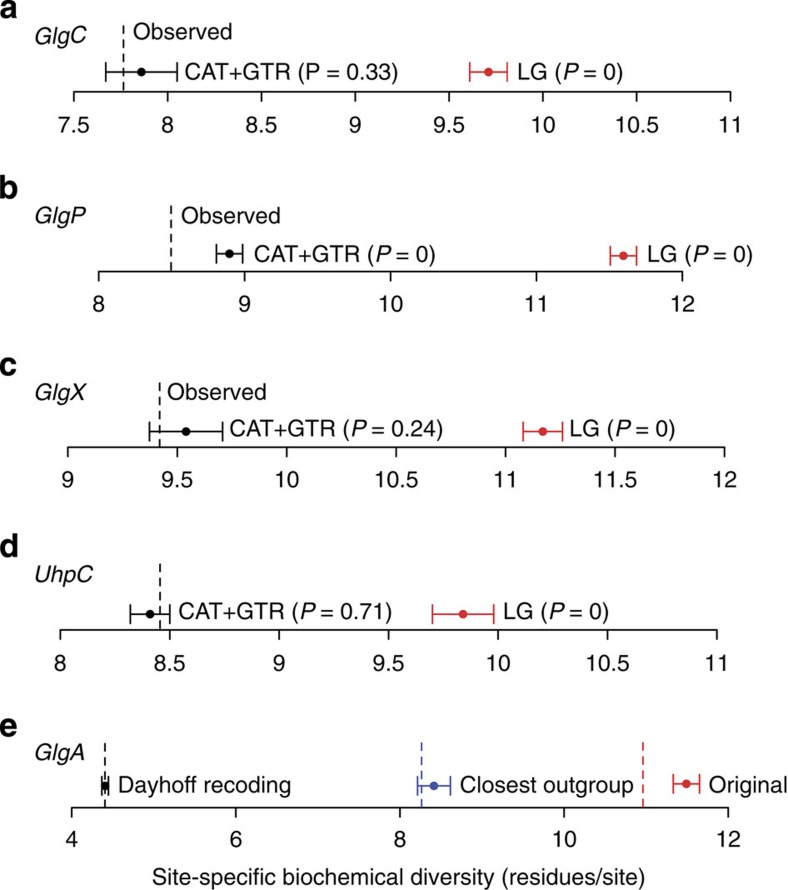
Bayesian posterior predictive simulations for assessing model fit to the key enzymes implicated in the ménage à trois. Posterior predictive simulations^25^ are a technique for assessing model adequacy with respect to key properties of the sequence alignment, which has an impact on phylogenetic inference. Here we compared the ability of the LG and CAT+GTR models to adequately capture the site-specific biochemical constraints experienced by the genes implicated in the ‘ménage à trois’ hypothesis. In sequence alignments, these constraints are manifest in the reduced number of amino acids observed in any one alignment column, which is usually much less than the theoretical maximum of 20. (**a**) The mean observed number of different amino acids per site in the GlgC alignment was 7.78. Data simulated under the LG model showed mean per-site diversity values (dot) much higher than the real data, suggesting this model did a poor job of modelling site-specific constraints. In contrast, the range (bars) of site-specific diversities predicted under the CAT+GTR model was comparable to that of the real data (*P*=0.33), suggesting adequate model fit with respect to this important metric. (**b**–**d**) The results for our analyses of GlgP, GlgX and UhpC were similar, with the CAT+GTR model better able to capture site-specific constraints, although neither model produced realistic predictions for the GlgP alignment. (**e**) Analyses of three different GlgA alignments under the CAT+GTR model. The original data set contained a large and highly diverse outgroup, leading to a high per-site diversity and poor model fit. An outgroup consisting only of the sequences most closely related to the relevant GlgA clade reduced per-site diversity and enabled adequate model fit; Dayhoff recoding of the original alignment also resulted in improved model fit relative to the unrecoded data. In both analyses in which adequate model fit was achieved, we did not recover a specific *Chlamydiae*/Archaeplastida clade, as discussed in the main text. Error bars represent s.e. and *P*-values were calculated using the ‘ppred’ and ‘readpb_mpi’ programmes in the PhyloBayes and PhyloBayes-MPI packages, respectively.

Under the ménage à trois hypothesis, archaeplastidal GlgC, GlgA, GlgP and GlgX originated as chlamydial effectors whose coding sequences were later transferred to the host nucleus; as a consequence, their modern-day archaeplastidal homologues are expected to cluster within, or as the sister to, chlamydial genes in single gene trees. Published phylogenies of these genes have made use of the single-matrix substitution models JTT^13^, WAG^14^,^15^,^23^ and LG^16^, which all share the simplifying assumptions that the process of evolution is homogeneous across the sites of the alignment and the branches of the tree. These assumptions are frequently violated by real molecular sequences, in which sequence composition, and by inference evolutionary process, often varies extensively in both of these dimensions. Violation of these assumptions results in poor model fit and can lead to phylogenetic artefacts such as long-branch attraction, in which fast-evolving sequences (long branches) cluster together irrespective of true historical relationships; as a result, analyses with poorly fitting models can potentially lead to the recovery of strongly supported but incorrect phylogenetic trees^24^. To evaluate whether the assumptions of single-matrix models are met by the enzymes key to the ménage à trois hypothesis, we performed posterior predictive simulations^25^ on alignments of GlgC, GlgX, GlgA, GlgP and UhpC under the LG model, which according to analyses using the model selection tool ProtTest 3.4 (ref. 26) was the best-fitting single-matrix model in all cases. Posterior predictive simulations provide a test of model adequacy by comparing the properties of data simulated under the model to the real alignment; significant compositional differences between the observed and simulated data suggest that the assumptions of the model are unrealistic for the data at hand. Our simulations indicated that all five alignments contained significant across-site and across-branch compositional variation that was not adequately accounted for by the single-matrix LG model (see Fig. 2 and Supplementary Table 1). These results suggested that LG provided an inadequate fit to the data with respect to sequence composition, raising the possibility that the resulting phylogenies might be affected by phylogenetic artefacts such as long-branch attraction and motivating the use of more complex models.

### Better-fitting models do not support the ménage à trois

In the last decade, growing recognition of the problems of systematic error in phylogenetics^24^, improvements in computational power and the increasing popularity of Bayesian approaches have stimulated the development of more complex phylogenetic models that can accommodate across-site and across-branch compositional variation^27^,^28^,^29^. These are pervasive features of real sequence data that, when not adequately modelled, are known to lead to topological errors in inferred trees. In particular, variation in sequence composition across the sites of an alignment is a ubiquitous feature of sequence data that arises from the site-specific selective constraints experienced by functional biological molecules; failure to account for the impact of these constraints on sequence evolution often results in poor modelling of the substitution process and can lead to phylogenetic artefacts such as long-branch attraction (LBA)^24^. One of the most useful approaches to modelling these site-specific constraints is the CAT family of substitution models^29^ that accommodate across-site compositional variation by allowing sites to be fit by distinct equilibrium composition profiles; as a result, these models have been shown to be more resistant that standard single-matrix models to systematic phylogenetic error and LBA^30^. We therefore applied these methods to the archaeplastidal genes predicted to trace their ancestry to the chlamydial partner in the ‘ménage à trois’. We compared the fit of these more complex models to the single-matrix models previously applied to these genes using posterior predictive simulations; the results of these tests are summarized in Fig. 2 and Supplementary Table 1, and are discussed on a per-gene basis below.

**Figure 3:**
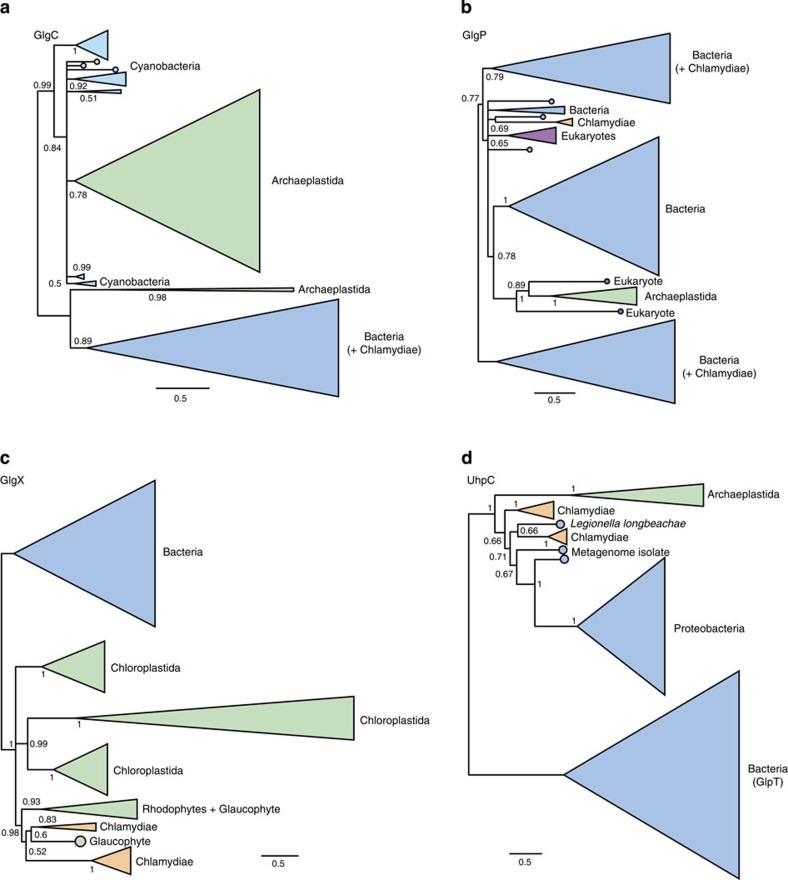
Single gene trees for key components of archaeplastidal carbohydrate metabolism implicated in the ménage à trois. (**a**–**d**) Phylogenies for GlgC, GlgP, GlgX and UhpC. These trees were inferred under the CAT+GTR model in PhyloBayes, which performed better in our analyses of model fit than the single-matrix models originally used to analyse these genes. With the exception of the *Chlamydomonas* and *Ostreococcus* GlgC sequences, the Archaeplastida were recovered as a monophyletic group in all of these trees, suggesting that this pathway was already present in its current form in the last common ancestor of the group. However, the closest outgroup to the Archaeplastida varies among the individual gene trees, as discussed in the main text. We rooted the tree in panel (**d**) between UhpC and its paralogue GlpT. In the other panels, we oriented the trees to most clearly visualize the key relationships between the archaeplastid and chlamydial sequences, and to test the predictions of the ménage à trois hypothesis. Support values are summarized as Bayesian posterior probabilities and branch lengths are proportional to the expected number of substitutions per site, as indicated by the scale bar.

The first step in manipulation of the heterotrophic host cell by the ancient chlamydial pathogen is suggested to be the conversion of host energy, in the form of glucose-1-phosphate, to ADP-glucose via the ADP-pyrophosphorylase GlgC. However, our phylogenetic analyses of GlgC homologues from Archaeplastida, *Chlamydiaceae*, *Cyanobacteria* and other bacterial groups recovered the archaeplastid sequences clustering with the *Cyanobacteria* with maximal posterior support (Posterior probability, PP=0.99 in the CAT+GTR analysis; see Fig. 3a and Supplementary Fig. 1). Within this clade, the archaeplastid sequences (with the exception of those from the green algae *Chlamydomonas* and *Ostreococcus*) emerged from within the *Cyanobacteria*, albeit with more modest support (PP=0.84). The simplest interpretation of these results is that glgC of modern Archaeplastida was obtained directly from the cyanobiont by endosymbiotic gene transfer^31^.

Following the generation of ADP-glucose by GlgC and its incorporation into host glycogen by GlgA (our analysis of which is discussed below), the next step in the exploitation of host energy by the ancient chlamydial pathogen is proposed to be the priming of glycogen for attack by parasite isoamylase (GlgX)^16^. The enzyme that performs this step is a glycogen phosphorylase, GlgP, which catalyses glycogen breakdown by recessing glycogen chains to within four residues of an α-1,6 branch. Our phylogenetic analyses did not recover a chlamydial, or indeed a cyanobacterial, origin for archaeplastidal glgP under any of the models used. Instead, the archaeplastidal sequences grouped with some other eukaryotes away from both the cyanobacterial and chlamydial clades (Fig. 3b and Supplementary Fig. 2), consistent with vertical descent of GlgP from the heterotrophic host cell for the cyanobacterial endosymbiont.

Under the ménage à trois hypothesis, the original role of chlamydial GlgX (isoamylase) was the generation of maltotetraose from host glycogen for import into the pathogen. Published trees inferred under the LG model^16^ recovered a *Chlamydiae/*archaeplastid clade clearly distinct from other bacteria, but support for the relationships within this clade were weak. Phylogenetic inference under the better-fitting CAT+GTR model (*P*=0.24 for across-site compositional heterogeneity, suggesting adequate model fit; see Fig. 2c and Supplementary Table 1) recovered a well-resolved *Chlamydiae*/archaeplastid clade in which the *Chlamydiae* emerged from within the Archaeplastida with high posterior support (PP=0.98 for CAT+GTR; see Fig. 3c and Supplementary Fig. 3). These results are consistent with the horizontal transfer of GlgX between *Chlamydiae* and Archaeplastida, but suggest that the direction was to, rather than from, the *Chlamydia* and hence they do not support the ménage à trois hypothesis.

### Origins of the UhpC hexose phosphate transporter

Genome analysis of the glaucophyte *Cyanophora paradoxa* has recently identified a homologue of UhpC, a hexose transporter of potential chlamydial origin^32^. This finding prompted a revision of the ménage à trois scenario in which an initial horizontal transfer of the *uhpC* gene from the chlamydial pathogen to the genome of the cyanobacterial endosymbiont provided the transporter needed for the export of photosynthate from the cyanobiont, in the form of glucose-1-phosphate^33^. It is worth pointing out that as the heterotrophic host cell would then have been able to make use of glucose-1-phosphate directly, this extension of the ménage à trois might seem to obviate the subsequent need for the chlamydial partner. Nonetheless, the revised theory locates both the cyanobiont and chlamydial partner in an inclusion within the eukaryotic host cell and posits that the glucose-1-phosphate exported from the cyanobiont by the chlamydial transporter was subsequently converted to ADP-glucose by chlamydial GlgC and transported into the host cytoplasm by a host-derived transporter, where it then followed the same fate as in the original ménage à trois model^33^.

A key issue when inferring and interpreting phylogenies of multigene families is the placement of the root, in particular for a transporter such as UhpC that is a member of the major facilitator superfamily and is related to the glycerol-3-phosphate transporter GlpT^34^. The published tree^32^ includes UhpC sequences from *Proteobacteria*, *Chlamydiae* and Archaeplastida and, when rooted on or within the *Proteobacteria*, produces a topology consistent with horizontal transfer of the gene from *Chlamydiae* to Archaeplastida. Our UhpC phylogeny, inferred under the better-fitting CAT+GTR model, would also support a transfer from *Chlamydiae* into plants but only if it too was rooted within the proteobacteria. By contrast, inclusion of the GlpT sequences as an outgroup placed the root between the archaeplastidal and bacterial sequences as a whole (Fig. 3d and Supplementary Fig. 4), eliminating any compelling case for specific gene transfer from *Chlamydiae* to Archaeplastida.

### High levels of compositional heterogeneity in GlgA

**Figure 4:**
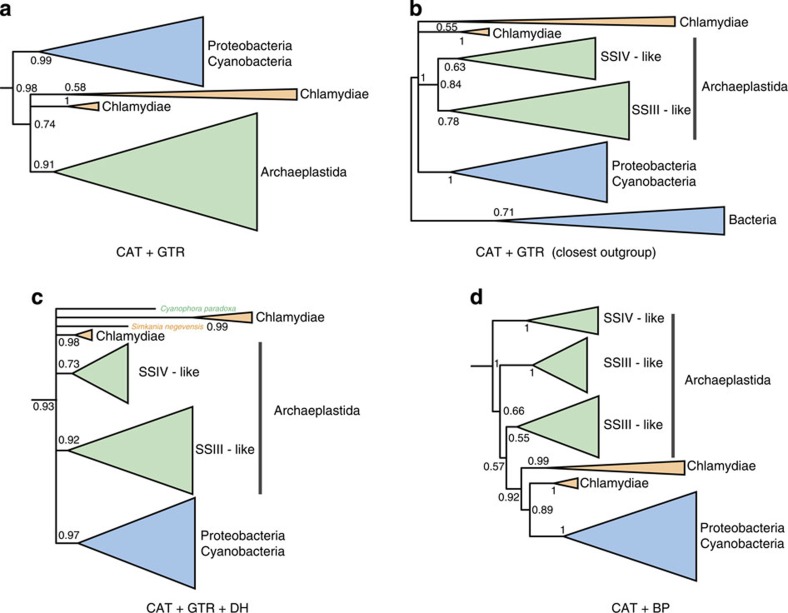
Phylogenetic analyses of the glycogen synthase GlgA. (**a**) Inference under the CAT+GTR model recovers a weakly supported (PP=0.74) clade comprising the chlamydial and Archaeplastidal sequences, but does not support horizontal transfer from *Chlamydiae* to Archaeplastida. This alignment was unusually heterogeneous in terms of sequence composition, and the CAT+GTR model failed our posterior predictive test for across-site compositional heterogeneity (*P*=0). (**b**) Inclusion of only the closest outgroup sequences improved the fit of the CAT+GTR model and collapsed this relationship, recovering an in-group trichotomy between the sequences from Archaeplastida, *Chlamydiae* and other bacteria. (**c**) Analysis of the Dayhoff-recoded data set under the CAT+GTR model; Dayhoff recoding ameliorated the observed compositional heterogeneity and also failed to recover a specific *Chlamydiae*/Archaeplastida relationship. (**d**) Joint modelling of across-site and across-branch compositional variation using the non-stationary CAT+BP model, which also failed to recover a specific relationship. These panels represent sub-trees derived from larger analyses showing the portion of the tree containing the chlamydial and archaeplastidal sequences; the root positions indicated are based on the topology of the complete analyses. Support values are summarized as Bayesian posterior probabilities, and branch lengths are proportional to the expected number of substitutions per site.

In the ménage à trois, the original role of the glycogen synthase GlgA is proposed to have been the incorporation of ADP-glucose generated by GlgC into host glycogen for later exploitation by the chlamydial pathogen. This enzyme would therefore have established the intial link between host and cyanobiont metabolism by providing a route for the incorporation of a bacterial metabolite (ADP-glucose) into host energy stores. In agreement with the recent analyses of Ball *et al*.^16^, a phylogeny inferred under the LG model provides moderate support (PP=0.83) for chlamydial ancestry of the archaeplastid sequences, although this model was rejected in our posterior predictive simulations both for across-site and across-branch compositional heterogeneity (*P*=0 for across-site compositional heterogeneity, *P*=0.002 for across-branch heterogeneity; see Supplementary Table 1). Indeed, the GlgA alignment proved to be extremely heterogeneous in both across-site and across-branch composition; unusually, even the most general substitution model currently available (CAT+GTR) failed to provide an adequate fit with respect to across-site compositional variation (*P*=0, see Fig. 2e and Supplementary Table 1). Although the tree inferred under CAT+GTR did not fit the data, it did weakly (PP=0.74) support a *Chlamydiae* plus Archaeplastida clade, consistent with horizontal exchange (Fig. 4a and Supplementary Fig. 5).

The original GlgA alignment of Ball *et al*.^16^ contains, in addition to the chlamydial and archaeplastidal sequences that are key to the ménage à trois hypothesis, an extensively sampled outgroup that includes distantly related, functionally divergent paralogues of these enzymes from Archaeplastida and bacteria. We reasoned that the large evolutionary distances, functional shifts and associated long branches that characterize this outgroup might be a contributing factor to the failure of the CAT+GTR model to adequately capture the compositional heterogeneity evident in the data set, potentially interfering with the inference of in-group relationships^35^. To test this idea, we removed most of the outgroup sequences, retaining only the clade that branched closest to the key *Chlamydiae*/Archaeplastida clade in the initial CAT+GTR analysis, and performed inference on this reduced data set under the same model. The removal of the more distant outgroup sequences significantly reduced the compositional heterogeneity in the data set so that the CAT+GTR model now provided an adequate fit for across-site compositional variation (*P*=0.21, see Fig. 2e), although it still failed the across-branch test (see Supplementary Table 1). Interestingly, this analysis no longer recovered a specific *Chlamydiae*/Archaeplastida clade (Fig. 4b and Supplementary Fig. 6), suggesting that the weakly supported relationship observed in the original tree may have been the result of poor model fit.

Given the poor fit of CAT+GTR to the GlgA sequences, we also evaluated two alternative approaches for modelling the evolution of GlgA: Dayhoff recoding and joint modelling of across-site and across-branch compositional variation using the CAT+BP model. In Dayhoff recoding^36^, the 20 amino acids are clustered into 6 bins such that the substitution rates among amino acids in the same bin are higher than between bins. By recoding amino acid data into these six classes and only modelling substitutions between bins, the degree of substitutional saturation and compositional heterogeneity in the data is greatly reduced, often helping to ameliorate poor model fit and the effects of systematic phylogenetic error^37^. Dayhoff recoding inevitably results in some information loss, but the net effect is often a substantial improvement in phylogenetic accuracy^37^, especially on extremely heterogeneous data sets such as the GlgA alignment^16^ that we analyse here. Indeed, posterior predictive tests on the complete alignment of Ball *et al*.^16^ analysed under the CAT+GTR model showed adequate fit with respect to both across-site and across-branch composition after Dayhoff recoding (*P*=0.49 and 0.1, respectively; see Fig. 2e and Supplementary Table 1), demonstrating a large improvement in model fit over the un-recoded data. As with our analysis on the unrecoded data using only the closest outgroup, the phylogeny inferred under this model did not recover a specific *Chlamydiae*/Archaeplastida relationship (Fig. 4c and Supplementary Fig. 7), instead recovering a clade (PP=0.93) in which the relationships among the *Chlamydiae*, Archaeplastida SSIII and SSIV-like, and other bacterial sequences were unresolved.

Finally, we attempted to jointly model the across-branch and across-site compositional variation in the GlgA alignment using the non-stationary CAT+BP model^27^, which combines modelling of across-site composition in the same way as the CAT model with a process in which composition can change at breakpoints (BPs) across the phylogenetic tree, leading to across-branch compositional variation. These analyses also failed to recover a *Chlamydiae*/Archaeplastida clade (Fig. 4d and Supplementary Fig. 8). Overall, our analyses demonstrate that the evolution of the GlgA gene is unusually difficult to model, given the high levels of both across-site and across-branch compositional variation observed. Nonetheless, our analyses using a series of better-fitting models suggest that there is no convincing support for a specific *Chlamydiae*/Archaeplastida relationship.

In summary, our phylogenetic analyses suggest a mosaic origin for archaeplastidal carbohydrate metabolism: the ADP-pyrophosphorylase GlgC descends from within the cyanobacteria, consistent with an origin from the cyanobacterial endosymbiont; the glycogen phosphorylase GlgP may descend from the eukaryotic host cell for that endosymbiont, and the glycogen synthase GlgA, the debranching enzyme GlgX and the hexose phosphate transporter UhpC appear to have bacterial, but not necessarily chlamydial, origins. Thus, in contrast to the predictions of the ménage à trois hypothesis, our analyses suggest that there is no compelling evidence that any of the key genes of archaeplastidal carbohydrate metabolism were acquired from an ancient chlamydial partner.

### Implications for the plastid endosymbiosis

In addition to the genes directly implicated in the ménage à trois hypothesis that we discuss above, support for chlamydial involvement in the establishment of the plastid has also been derived from the observation that nearly 60 archaeplastidal genes group with *Chlamydiae* in genomic surveys of single-gene trees^12^,^13^,^14^,^15^,^16^,^38^. These trees have been interpreted as evidence of a batch horizontal transfer from *Chlamydiae* to Archaeplastida that could also reflect a long period of infection, symbiosis or co-habitation of the same ecological niche^13^,^14^,^16^. For reasons of computational speed, phylogenomic screens have employed single-matrix methods, such as the LG model discussed above, and are therefore subject to the same caveats as the gene trees analysed here. Beyond these methodological concerns, there is a deeper problem with inferring a special explanation for the presence of putative chlamydial genes on plant genomes, in the absence of any physical evidence of the proposed chlamydial partner. The problem is that recent studies have demonstrated that in addition to organellar genes shared with *Cyanobacteria* and *Alphaproteobacteria*, the Archaeplastida share more genes with *Gammaproteobacteria*, *Actinobacteria*, *Deltaproteobacteria*, *Bacilli*, *Bacteroidetes* and *Betaproteobacteria* than with *Chlamydiae*^8^. Given the extent of HGT, particularly of metabolic genes, among major cellular groups^39^ and the demonstrated limitations of standard phylogenetic models for the archaeplastidal genes we analysed here, these patterns of gene sharing—including those involving *Chlamydiae*—are most simply explained as a mixture of genuine HGT events and tree reconstruction artefacts. Thus, in the absence of cytological evidence for a *chlamydia*-derived organelle, or support for the ménage à trois hypothesis from better-fitting phylogenetic models, we conclude that there is no compelling need to invoke a chlamydial partner in the establishment of the primary plastid endosymbiosis.

## Methods

### Sequences and alignments

The GlgA and GlgX alignments were those used in Ball *et al*.^16^ For the other genes, gene families were downloaded from the HOGENOM^40^ (UhpC and GlgC) or OMA^41^ (GlgP) databases and augmented with their orthologues from a set of newly sequenced chlamydial genomes (*Neochlamydia sp.* TUME1 and EPS4, *Protochlamydia sp.* EI2 and *Parachlamydia sp*. OEW1) as well as additional cyanobacterial orthologues. Sequences for GlgC and GlgP were aligned using Muscle 3.8 (ref. 42) and poorly aligning regions were detected and removed using BMGE^43^ with the BLOSUM30 scoring matrix. UhpC sequences were collected by extracting the top 250 BLAST hits of the *Protochlamydia amoebophila* homologue (Q6ME88_PARUW) against the UniRef90 database^44^ and supplemented with the aforementioned chlamydial genomes. The alignment was performed using clustalOmega^45^ and filtered using GBlocks^46^ by using the parameters ‘-b4=3 -b5=a’. All sequence sets, alignments and Newick tree files have been deposited in FigShare (http://dx.doi.org/10.6084/m9.figshare.1257740).

### Phylogenetic analyses

Analyses using the CAT+GTR and CAT+GTR+Dayhoff models were performed using PhyloBayes-MPI^47^ 1.5a and analyses using CAT-BP were performed using nhPhyloBayes^27^. Bayesian analyses using the LG model were performed in PhyloBayes 3.3 (ref. 48). For each analysis, two chains were run in parallel, and the bpcomp and tracecomp programmes were used to assess convergence. We judged that analyses had converged when the maximum discrepancies in bipartition frequencies (bpcomp) and summary statistics (tracecomp) between the two chains had all dropped below 0.1, and the effective sample size of each parameter was at least 100, as recommended in the PhyloBayes manual (http://www.phylobayes.org).

### Posterior predictive simulations

Posterior predictive simulations were performed using converged runs to evaluate model fit. We used the ppred (PhyloBayes 3.3) and readpb_mpi (PhyloBayes-MPI 1.5a) programmes to perform tests of across-site (site-specific biochemical diversity) and across-branch (compositional homogeneity) tests for the LG, CAT+GTR and CAT+GTR+Dayhoff models. We judged that a model failed a particular test if the test statistic calculated on the real data fell outside the central 95% of the simulated distribution.

## Additional information

**How to cite this article:** Domman, D. *et al*. Plastid establishment did not require a chlamydial partner. *Nat. Commun.* 6:6421 doi: 10.1038/ncomms7421 (2015).

## Supplementary Material

Supplementary Information Supplementary Figures 1-8 and Supplementary Table 1

## Figures and Tables

**Figure 1:**
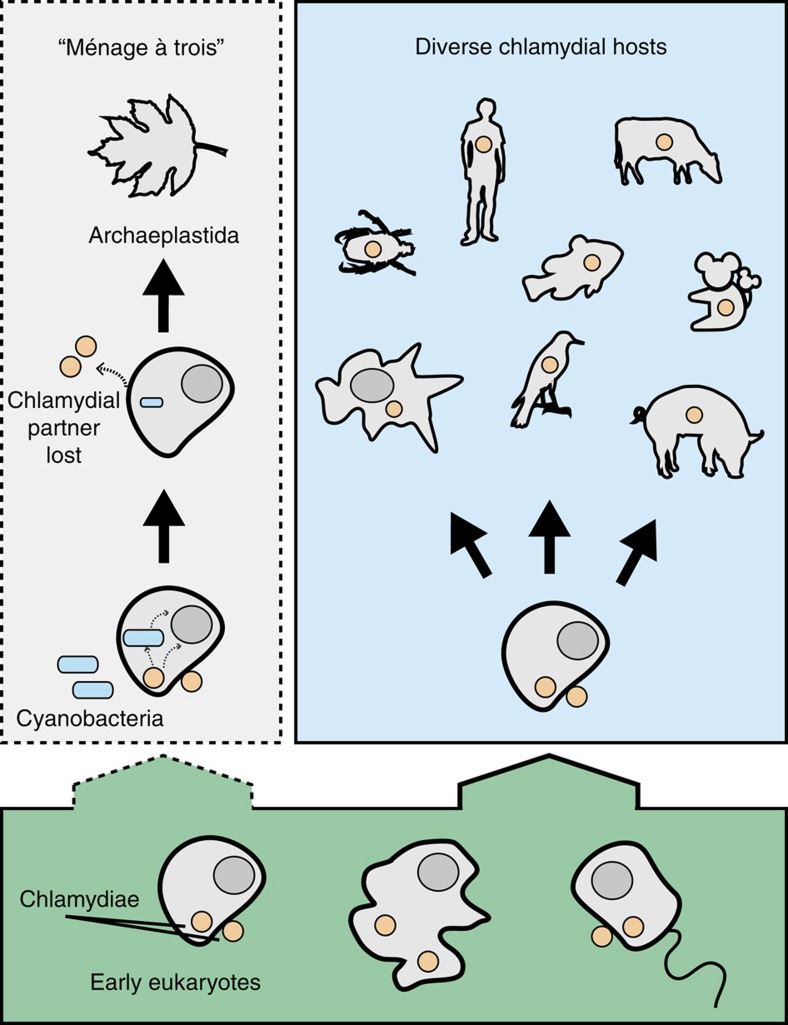
The evolutionary history of chlamydia–eukaryote interactions and the ménage à trois hypothesis for plastid establishment. Chlamydiae have probably been associated with eukaryotes for at least 700 million years (1717) so it appears reasonable to suggest that they also infected even more ancient eukaryotes. Extant *Chlamydiae* can infect a tremendously diverse range of eukaryotic hosts such as humans, cattle, pigs, birds, koala, fish, insects and unicellular protists. Notably, *Chlamydiae* have not been found infecting any member of the Archaeplastida. A proposed evolutionary scenario, coined the ‘ménage à trois’ hypothesis^16^, posits that an early eukaryotic cell was host to both a chlamydial and cyanobacterial partner. Key metabolic genes that enabled the symbiotic capture of the cyanobacterium are proposed to have been horizontally transferred from chlamydia primarily to the host, but also to the cyanobacterium. Once these genes were transferred, the chlamydial partner was no longer needed and was subsequently lost. The newly formed relationship between cyanobacterium and host led to the modern plastid and the evolution of Archaeplastida.
