# Supplementary material for: Plastid establishment did not require a chlamydial partner
Source: Nat Commun. 2015 Mar 11;6:6421. doi: 10.1038/ncomms7421 (PMC4374161; doi:10.1038/ncomms7421)
Supplement: Supplementary Information — Supplementary Figures 1-8 and Supplementary Table 1 [file ncomms7421-s1.pdf]

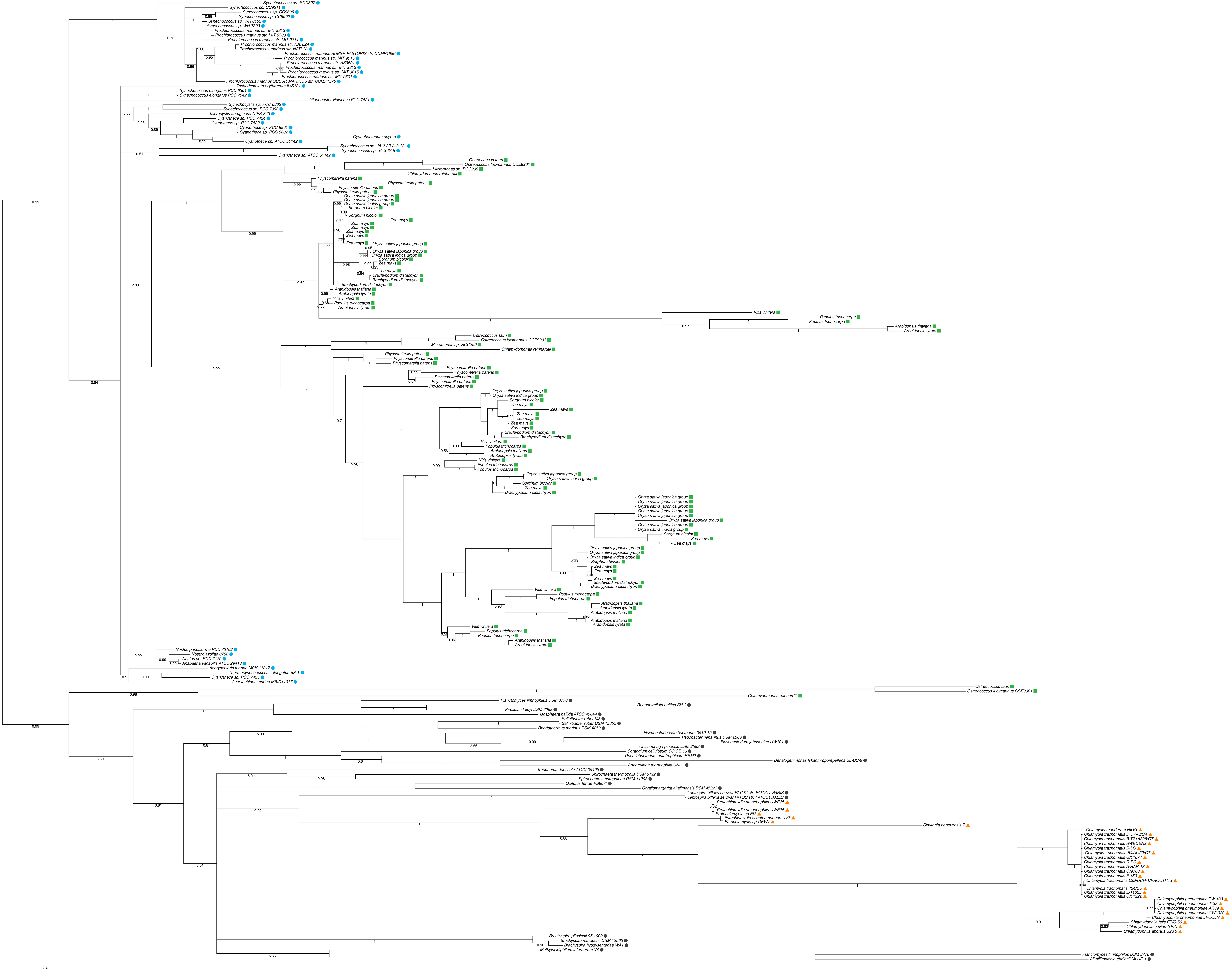
Synechococcus sp. RCC307
Synechococcus sp. CC9311
Synechococcus sp. CC9605
Synechococcus sp. CC9902
Synechococcus sp. WH 8102
Synechococcus sp. WH 7803
Prochlorococcus marinus str. MIT 9313
Prochlorococcus marinus str. MIT 9303
Prochlorococcus marinus str. MIT 9211
Prochlorococcus marinus str. NATL2A
Prochlorococcus marinus str. NATL1A
Prochlorococcus marinus SUBSP. PASTORIS str. CCMP1986
Prochlorococcus marinus str. MIT 9515
Prochlorococcus marinus str. AS9601
Prochlorococcus marinus str. MIT 9312
Prochlorococcus marinus str. MIT 9215
Prochlorococcus marinus str. MIT 9301
Prochlorococcus marinus SUBSP. MARINUS str. CCMP1375
Trichodesmium erythraeum IMS101
Synechococcus elongatus PCC 6301
Synechococcus elongatus PCC 7942
Gloeobacter violaceus PCC 7421
Synechocystis sp. PCC 6803
Synechococcus sp. PCC 7002
Microcystis aeruginosa NIES-843
Cyanothece sp. PCC 7424
Cyanothece sp. PCC 7822
Cyanothece sp. PCC 8801
Cyanothece sp. PCC 8802
Cyanobacterium ucyn-a
Cyanothece sp. ATCC 51142
Synechococcus sp. JA-2-3B'A.2-13.
Synechococcus sp. JA-3-3AB
Cyanothece sp. ATCC 51142
Ostreococcus tauri
Ostreococcus lucimarinus CCE9901
Micromonas sp. RCC299
Chlamydomonas reinhardtii
Physcomitrella patens
Oryza sativa japonica group
Oryza sativa indica group
Sorghum bicolor
Zea mays
Brachypodium distachyon
Arabidopsis thaliana
Arabidopsis lyrata
Vitis vinifera
Populus trichocarpa
Nostoc punctiforme PCC 73102
Nostoc azollae 0708
Nostoc sp. PCC 7120
Anabaena variabilis ATCC 29413
Acaryochloris marina MBIC11017
Thermosynechococcus elongatus BP-1
Cyanothece sp. PCC 7425
Planctomyces limnophilus DSM 3776
Rhodopirellula baltica SH 1
Pirellula staleyi DSM 6068
Isosphaera pallida ATCC 43644
Salinibacter ruber M8
Salinibacter ruber DSM 13855
Rhodothermus marinus DSM 4252
Flavobacteriaceae bacterium 3519-10
Pedobacter heparinus DSM 2366
Flavobacterium johnsoniae UW101
Chitinophaga pinensis DSM 2588
Sorangium cellulosum SO CE 56
Desulfobacterium autotrophicum HRM2
Dehalogenimonas lykanthroporepellens BL-DC-9
Anaerolinea thermophila UNI-1
Treponema denticola ATCC 35405
Spirochaeta thermophila DSM 6192
Spirochaeta smaragdinae DSM 11293
Opitutus terrae PB90-1
Coraliomargarita akajimensis DSM 45221
Leptospira biflexa serovar PATOC str. PATOC1.PARIS
Leptospira biflexa serovar PATOC1.AMES
Protochlamydia amoebophila UWE25
Protochlamydia sp EI2
Parachlamydia acanthamoebae UV7
Parachlamydia sp OEW1
Simkania negevensis Z
Chlamydia muridarum NIGG
Chlamydia trachomatis D/UW-3/CX
Chlamydia trachomatis B/TZ1A828/OT
Chlamydia trachomatis SWEDEN2
Chlamydia trachomatis D-LC
Chlamydia trachomatis B/JALI20/OT
Chlamydia trachomatis G/11074
Chlamydia trachomatis D-EC
Chlamydia trachomatis A/HAR-13
Chlamydia trachomatis G/9768
Chlamydia trachomatis E/150
Chlamydia trachomatis L2B/UCH-1/PROCTITIS
Chlamydia trachomatis 434/BU
Chlamydia trachomatis E/11023
Chlamydia trachomatis G/11222
Chlamydophila pneumoniae TW-183
Chlamydophila pneumoniae J138
Chlamydophila pneumoniae AR39
Chlamydophila pneumoniae CWL029
Chlamydophila pneumoniae LPCOLN
Chlamydophila felis FE/C-56
Chlamydophila caviae GPIC
Chlamydophila abortus S26/3
Brachyspira pilosicoli 95/1000
Brachyspira murdochii DSM 12563
Brachyspira hyodysenteriae WA1
Methylacidiphilum infernorum V4
Planctomyces limnophilus DSM 3776
Alkalilimnicola ehrlichii MLHE-1

Supplementary Figure 1: **Phylogenetic analysis of the GlgC gene under the CAT+GTR model.** This is the complete tree upon which Fig. 3(a) is based. Archaeplastida sequences are denoted with green squares, *Chlamydiae* with orange triangles, *Cyanobacteria* with cyan circles, and other bacterial groups with black circles. Branch supports are Bayesian posterior probabilities, and branch lengths are proportional to the expected number of substitutions per site, as indicated by the scale bar.

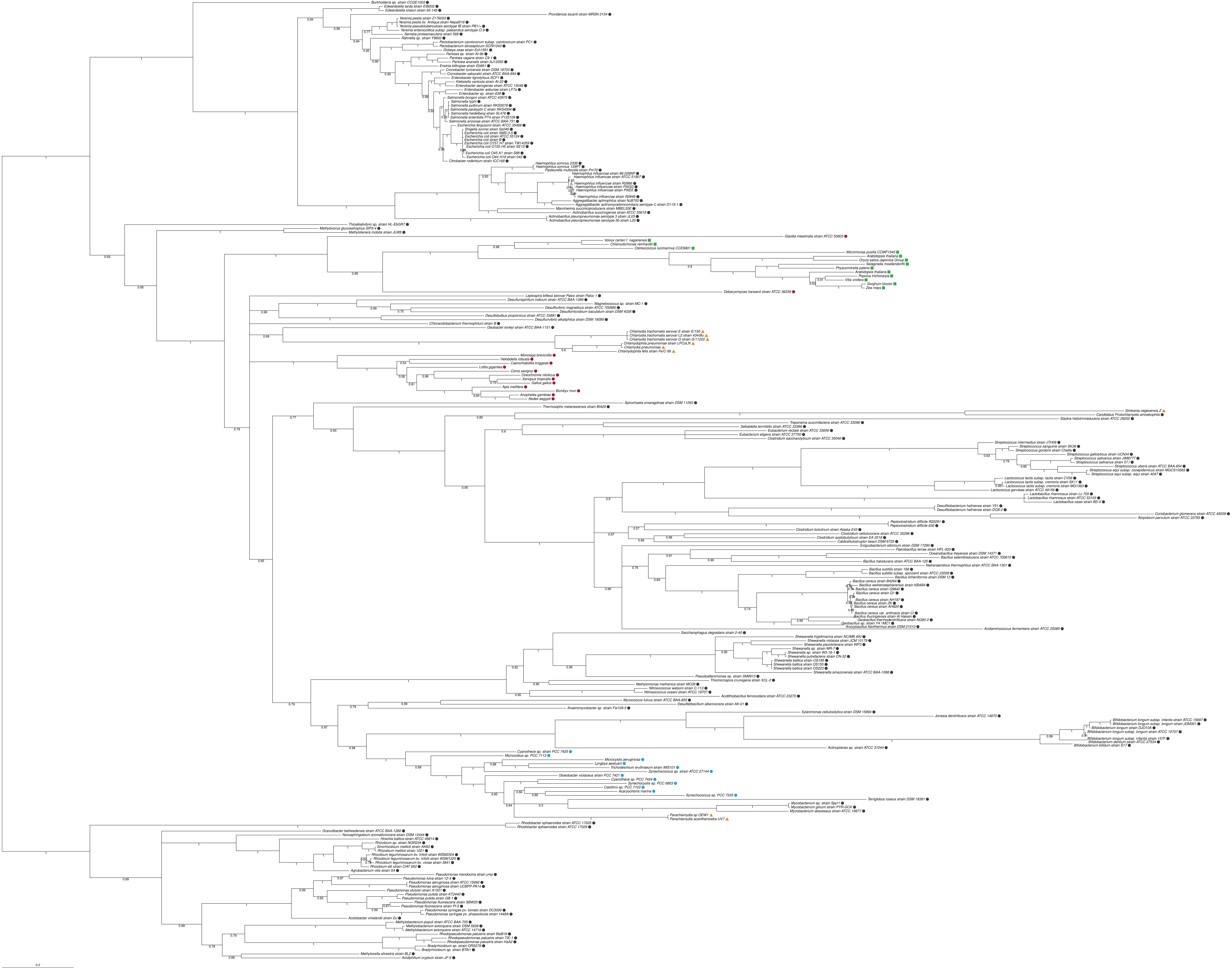

Burkholderia sp. strain CCGE1003
Edwardsiella tarda strain EIB202
Edwardsiella ictaluri strain 93-146
Providencia stuartii strain MRSN 2154
Yersinia pestis strain Z176003
Yersinia pestis bv. Antiqua strain Nepal516
Yersinia pseudotuberculosis serotype IB strain PB1/+
Yersinia enterocolitica subsp. palearctica serotype O:3
Serratia proteamaculans strain 568
Rahnella sp. strain Y9602
Pectobacterium carotovorum subsp. carotovorum strain PC1
Pectobacterium atrosepticum SCRI1043
Dickeya zeae strain Ech1591
Pantoea sp. strain At-9b
Pantoea vagans strain C9-1
Pantoea ananatis strain AJ13355
Erwinia billingiae strain Eb661
Cronobacter turicensis strain DSM 18703
Cronobacter sakazakii strain ATCC BAA-894
Enterobacter lignolyticus SCF1
Klebsiella variicola strain At-22
Enterobacter aerogenes strain ATCC 13048
Enterobacter asburiae strain LF7a
Enterobacter sp. strain 638
Salmonella bongori strain ATCC 43975
Salmonella typhi
Salmonella pullorum strain RKS5078
Salmonella paratyphi C strain RKS4594
Salmonella heidelberg strain SL476
Salmonella enteritidis PT4 strain P125109
Salmonella arizonae strain ATCC BAA-731
Escherichia fergusonii strain ATCC 35469
Shigella sonnei strain Ss046
Escherichia coli strain SMS-3-5
Escherichia coli strain ATCC 55124
Escherichia coli strain B
Escherichia coli O157:H7 strain TW14359
Escherichia coli O150:H5 strain SE15
Escherichia coli O45:K1 strain S88
Escherichia coli O44:H18 strain 042
Citrobacter rodentium strain ICC168
Haemophilus somnus 2336
Haemophilus somnus 129PT
Pasteurella multocida strain Pm70
Haemophilus influenzae strain 86-028NP
Haemophilus influenzae strain ATCC 51907
Haemophilus influenzae strain R2866
Haemophilus influenzae strain PittGG
Haemophilus influenzae strain PittEE
Haemophilus influenzae strain R2846
Aggregatibacter aphrophilus strain NJ8700
Aggregatibacter actinomycetemcomitans serotype C strain D11S-1
Mannheimia succiniciproducens strain MBEL55E
Actinobacillus succinogenes strain ATCC 55618
Actinobacillus pleuropneumoniae serotype 3 strain JL03
Actinobacillus pleuropneumoniae serotype 5b strain L20
Thioalkalivibrio sp. strain HL-EbGR7
Methylovorus glucosetrophus SIP3-4
Methylotenera mobilis strain JLW8
Giardia intestinalis strain ATCC 50803
Volvox carteri f. nagariensis
Chlamydomonas reinhardtii
Ostreococcus lucimarinus CCE9901
Micromonas pusilla CCMP1545
Arabidopsis thaliana
Oryza sativa Japonica Group
Selaginella moellendorffii
Physcomitrella patens
Arabidopsis thaliana
Populus trichocarpa
Vitis vinifera
Sorghum bicolor
Zea mays
Debaryomyces hansenii strain ATCC 36239
Leptospira biflexa serovar Patoc strain Patoc 1
Desulfurispirillum indicum strain ATCC BAA-1389
Magnetococcus sp. strain MC-1
Desulfovibrio magneticus strain ATCC 700980
Desulfomicrobium baculatum strain DSM 4028
Desulfobulbus propionicus strain ATCC 33891
Desulfurivibrio alkaliphilus strain DSM 19089
Chloracidobacterium thermophilum strain B
Geobacter lovleyi strain ATCC BAA-1151
Chlamydia trachomatis serovar E strain E/150
Chlamydia trachomatis serovar L2 strain 434/Bu
Chlamydia trachomatis serovar G strain G/11222
Chlamydophila pneumoniae strain LPCoLN
Chlamydia pneumoniae
Chlamydophila felis strain Fe/C-56
Monosiga brevicollis
Helobdella robusta
Caenorhabditis briggsae
Lottia gigantea
Ciona savignyi
Oreochromis niloticus
Xenopus tropicalis
Gallus gallus
Apis mellifera
Bombyx mori
Anopheles gambiae
Aedes aegypti
Spirochaeta smaragdinae strain DSM 11293
Thermosipho melanesiensis strain BI429
Simkania negevensis Z
Candidatus Protochlamydia amoebophila
Slackia heliotrinireducens strain ATCC 29202
Treponema succinifaciens strain ATCC 33096
Sebaldella termitidis strain ATCC 33386
Eubacterium rectale strain ATCC 33656
Eubacterium eligens strain ATCC 27750
Clostridium saccharolyticum strain ATCC 35040
Streptococcus intermedius strain JTH08
Streptococcus sanguinis strain SK36
Streptococcus gordonii strain Challis
Streptococcus gallolyticus strain UCN34
Streptococcus salivarius strain JIM8777
Streptococcus salivarius strain 57.I
Streptococcus uberis strain ATCC BAA-854
Streptococcus equi subsp. zooepidemicus strain MGCS10565
Streptococcus equi subsp. equi strain 4047
Lactococcus lactis subsp. lactis strain CV56
Lactococcus lactis subsp. cremoris strain SK11
Lactococcus lactis subsp. cremoris strain MG1363
Lactococcus garvieae strain ATCC 49156
Lactobacillus rhamnosus strain Lc 705
Lactobacillus rhamnosus strain ATCC 53103
Lactobacillus casei strain BD-II
Desulfitobacterium hafniense strain Y51
Desulfitobacterium hafniense strain DCB-2
Coriobacterium glomerans strain ATCC 49209
Atopobium parvulum strain ATCC 33793
Peptoclostridium difficile R20291
Peptoclostridium difficile 630
Clostridium botulinum strain Alaska E43
Clostridium cellulovorans strain ATCC 35296
Clostridium acetobutylicum strain EA 2018
Caldicellulosiruptor bescii DSM 6725
Exiguobacterium sibiricum strain DSM 17290
Paenibacillus terrae strain HPL-003
Oceanobacillus iheyensis strain DSM 14371
Bacillus selenitireducens strain ATCC 700615
Bacillus halodurans strain ATCC BAA-125
Natranaerobius thermophilus strain ATCC BAA-1301
Bacillus subtilis strain 168
Bacillus subtilis subsp. spizizenii strain ATCC 23059
Bacillus licheniformis strain DSM 13
Bacillus cereus strain B4264
Bacillus weihenstephanensis strain KBAB4
Bacillus cereus strain G9842
Bacillus cereus strain Q1
Bacillus cereus strain AH187
Bacillus cereus strain ZK
Bacillus cereus strain AH820
Bacillus cereus var. anthracis strain CI
Bacillus thuringiensis strain Al Hakam
Geobacillus thermodenitrificans strain NG80-2
Geobacillus sp. strain Y4.1MC1
Anoxybacillus flavithermus strain DSM 21510
Acidaminococcus fermentans strain ATCC 25085
Saccharophagus degradans strain 2-40
Shewanella frigidimarina strain NCIMB 400
Shewanella violacea strain JCM 10179
Shewanella piezotolerans strain WP3
Shewanella sp. strain MR-7
Shewanella sp. strain W3-18-1
Shewanella putrefaciens strain CN-32
Shewanella baltica strain OS185
Shewanella baltica strain OS155
Shewanella baltica strain OS223
Shewanella amazonensis strain ATCC BAA-1098
Pseudoalteromonas sp. strain SM9913
Thiomicrospira crunogena strain XCL-2
Methylomonas methanica strain MC09
Nitrosococcus watsoni strain C-113
Nitrosococcus oceani strain ATCC 19707
Acidithiobacillus ferrooxidans strain ATCC 23270
Myxococcus fulvus strain ATCC BAA-855
Desulfatibacillum alkenivorans strain AK-01
Anaeromyxobacter sp. strain Fw109-5
Xylanimonas cellulosilytica strain DSM 15894
Jonesia denitrificans strain ATCC 14870
Bifidobacterium longum subsp. infantis strain ATCC 15697
Bifidobacterium longum subsp. longum strain JDM301
Bifidobacterium longum strain DJO10A
Bifidobacterium longum subsp. longum strain ATCC 15707
Bifidobacterium longum subsp. infantis strain 157F
Bifidobacterium dentium strain ATCC 27534
Bifidobacterium bifidum strain S17
Actinoplanes sp. strain ATCC 31044
Cyanothece sp. strain PCC 7425
Microcoleus sp. PCC 7113
Microcystis aeruginosa
Lyngbya aestuarii
Trichodesmium erythraeum strain IMS101
Synechococcus sp. strain ATCC 27144
Gloeobacter violaceus strain PCC 7421
Cyanothece sp. PCC 7424
Synechocystis sp. PCC 6803
Calothrix sp. PCC 7103
Acaryochloris marina
Synechococcus sp. PCC 7335
Terriglobus roseus strain DSM 18391
Mycobacterium sp. strain Spyr1
Mycobacterium gilvum strain PYR-GCK
Mycobacterium abscessus strain ATCC 19977
Parachlamydia sp OEW1
Parachlamydia acanthamoeba UV7
Rhodobacter sphaeroides strain ATCC 17025
Rhodobacter sphaeroides strain ATCC 17029
Granulibacter bethesdensis strain ATCC BAA-1260
Novosphingobium aromaticivorans strain DSM 12444
Hirschia baltica strain ATCC 49814
Rhizobium sp. strain NGR234
Sinorhizobium meliloti strain AK83
Rhizobium meliloti strain 1021
Rhizobium leguminosarum bv. trifolii strain WSM2304
Rhizobium leguminosarum bv. trifolii strain WSM1325
Rhizobium leguminosarum bv. viciae strain 3841
Rhizobium etli strain CIAT 652
Agrobacterium vitis strain S4
Pseudomonas mendocina strain ymp
Pseudomonas fulva strain 12-X
Pseudomonas aeruginosa strain ATCC 15692
Pseudomonas aeruginosa strain UCBPP-PA14
Pseudomonas stutzeri strain A1501
Pseudomonas putida strain KT2440
Pseudomonas putida strain GB-1
Pseudomonas fluorescens strain SBW25
Pseudomonas fluorescens strain Pf-5
Pseudomonas syringae pv. tomato strain DC3000
Pseudomonas syringae pv. phaseolicola strain 1448A
Azotobacter vinelandii strain DJ
Methylobacterium populi strain ATCC BAA-705
Methylobacterium extorquens strain DSM 5838
Methylobacterium extorquens strain ATCC 14718
Rhodopseudomonas palustris strain BisB18
Rhodopseudomonas palustris strain TIE-1
Rhodopseudomonas palustris strain HaA2
Bradyrhizobium sp. strain ORS278
Bradyrhizobium sp. strain BTAi1
Methylocella silvestris strain BL2
Acidiphilium cryptum strain JF-5
0.2

Supplementary Figure 2: **Phylogenetic analysis of the GlgP gene under the CAT+GTR model.** This is the complete tree upon which Fig. 3(b) is based. Archaeplastida sequences are denoted with green squares, other eukaryotes with purple circles, *Chlamydiae* with orange triangles, *Cyanobacteria* with cyan circles, and other bacterial groups with black circles. Branch supports are Bayesian posterior probabilities, and branch lengths are proportional to the expected number of substitutions per site, as indicated by the scale bar.

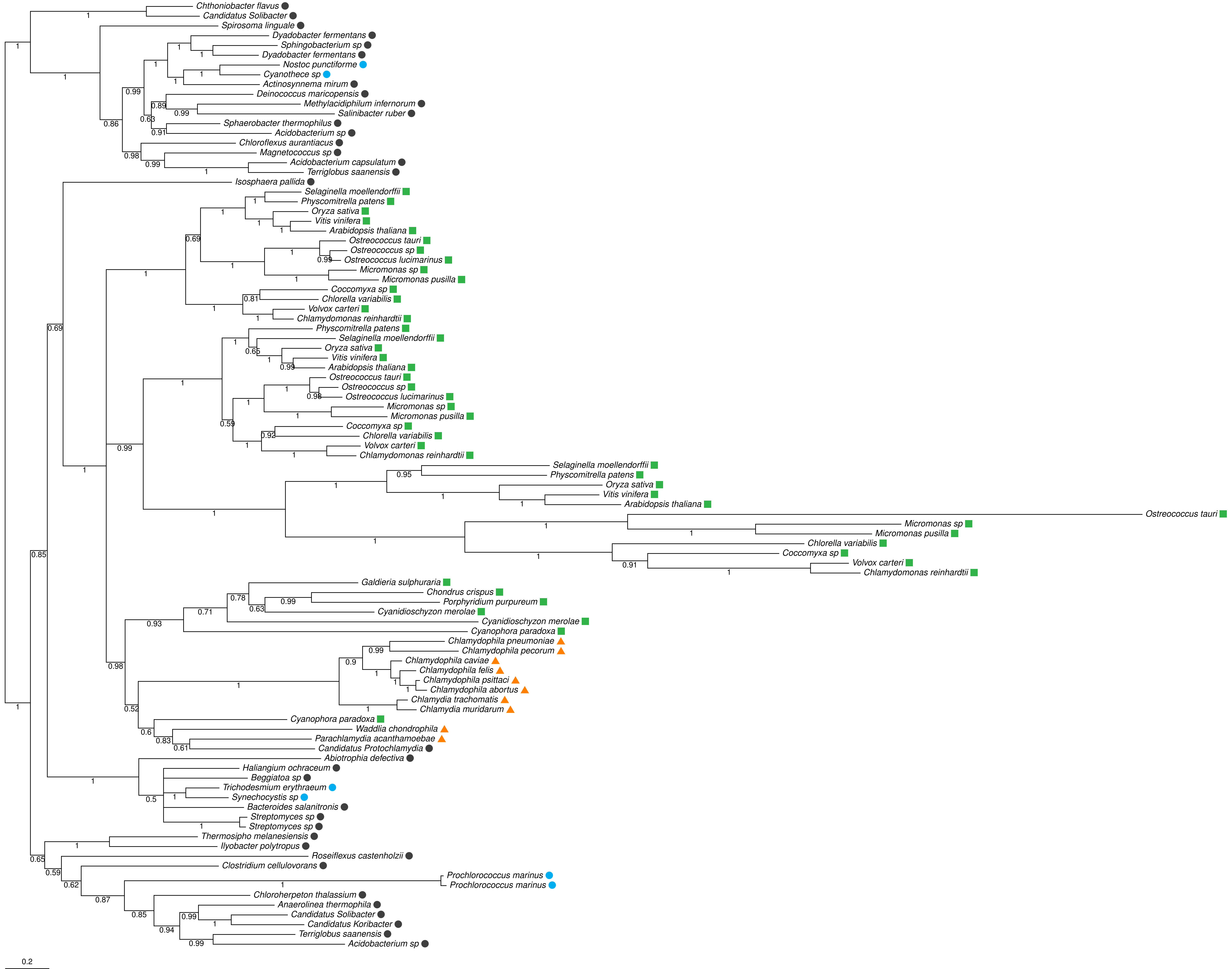

Chthoniobacter flavus
Candidatus Solibacter
Spirosoma linguale
Dyadobacter fermentans
Sphingobacterium sp
Dyadobacter fermentans
Nostoc punctiforme
Cyanothece sp
Actinosynnema mirum
Deinococcus maricopensis
Methylacidiphilum infernorum
Salinibacter ruber
Sphaerobacter thermophilus
Acidobacterium sp
Chloroflexus aurantiacus
Magnetococcus sp
Acidobacterium capsulatum
Terriglobus saanensis
Isosphaera pallida
Selaginella moellendorffii
Physcomitrella patens
Oryza sativa
Vitis vinifera
Arabidopsis thaliana
Ostreococcus tauri
Ostreococcus sp
Ostreococcus lucimarinus
Micromonas sp
Micromonas pusilla
Coccomyxa sp
Chlorella variabilis
Volvox carteri
Chlamydomonas reinhardtii
Physcomitrella patens
Selaginella moellendorffii
Oryza sativa
Vitis vinifera
Arabidopsis thaliana
Ostreococcus tauri
Ostreococcus sp
Ostreococcus lucimarinus
Micromonas sp
Micromonas pusilla
Coccomyxa sp
Chlorella variabilis
Volvox carteri
Chlamydomonas reinhardtii
Selaginella moellendorffii
Physcomitrella patens
Oryza sativa
Vitis vinifera
Arabidopsis thaliana
Ostreococcus tauri
Micromonas sp
Micromonas pusilla
Chlorella variabilis
Coccomyxa sp
Volvox carteri
Chlamydomonas reinhardtii
Galdieria sulphuraria
Chondrus crispus
Porphyridium purpureum
Cyanidioschyzon merolae
Cyanidioschyzon merolae
Cyanophora paradoxa
Chlamydophila pneumoniae
Chlamydophila pecorum
Chlamydophila caviae
Chlamydophila felis
Chlamydophila psittaci
Chlamydophila abortus
Chlamydia trachomatis
Chlamydia muridarum
Cyanophora paradoxa
Waddlia chondrophila
Parachlamydia acanthamoebae
Candidatus Protochlamydia
Abiotrophia defectiva
Haliangium ochraceum
Beggiatoa sp
Trichodesmium erythraeum
Synechocystis sp
Bacteroides salanitronis
Streptomyces sp
Streptomyces sp
Thermosipho melanesiensis
Ilyobacter polytropus
Roseiflexus castenholzii
Clostridium cellulovorans
Prochlorococcus marinus
Prochlorococcus marinus
Chloroherpeton thalassium
Anaerolinea thermophila
Candidatus Solibacter
Candidatus Koribacter
Terriglobus saanensis
Acidobacterium sp
0.2

Supplementary Figure 3: **Phylogenetic analysis of the GlgX gene under the CAT+GTR model.** This is the complete tree upon which Fig. 3(c) is based. Archaeplastida sequences are denoted with green squares, *Chlamydiae* with orange triangles, *Cyanobacteria* with cyan circles, and other bacterial groups with black circles. Branch supports are Bayesian posterior probabilities, and branch lengths are proportional to the expected number of substitutions per site, as indicated by the scale bar.

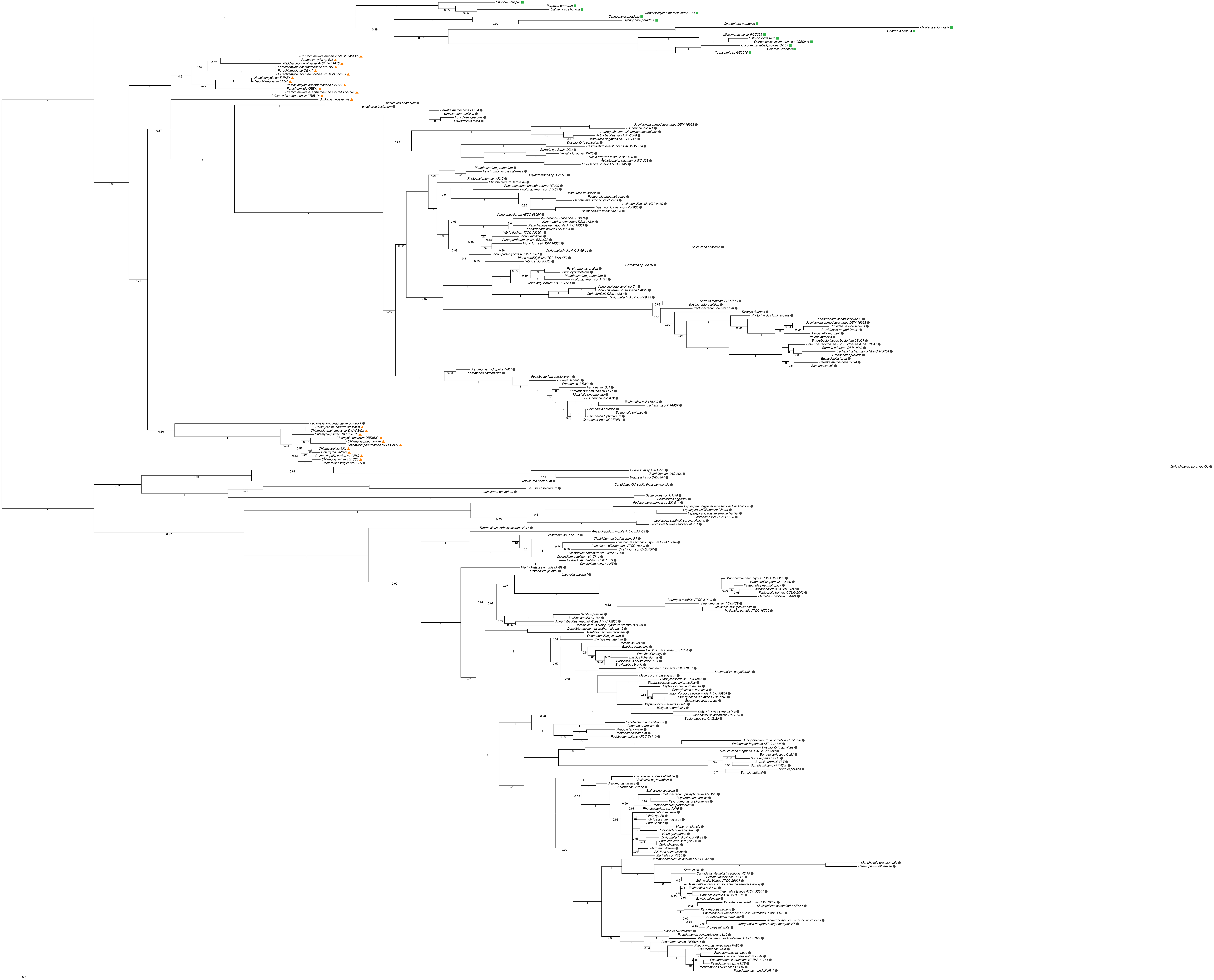

Chondrus crispus
Porphyra purpurea
Galdieria sulphuraria
Cyanidioschyzon merolae strain 10D
Cyanophora paradoxa
Cyanophora paradoxa
Cyanophora paradoxa
Galdieria sulphuraria
Chondrus crispus
Micromonas sp str RCC299
Ostreococcus tauri
Ostreococcus lucimarinus str CCE9901
Coccomyxa subellipsoidea C-169
Chlorella variabilis
Tetraselmis sp GSL018
Protochlamydia amoebophila str UWE25
Protochlamydia sp EI2
Waddlia chondrophila str ATCC VR-1470
Parachlamydia acanthamoebae str UV7
Parachlamydia sp OEW1
Parachlamydia acanthamoebae str Hall's coccus
Neochlamydia sp TUME1
Neochlamydia sp EPS4
Parachlamydia acanthamoebae str UV7
Parachlamydia OEW1
Parachlamydia acanthamoebae str Hall's coccus
Criblamydia sequanensis CRIB-18
Simkania negevensis
uncultured bacterium
uncultured bacterium
Serratia marcescens FGI94
Yersinia enterocolitica
Lonsdalea quercina
Edwardsiella tarda
Providencia burhodogranariea DSM 19968
Escherichia coli N1
Aggregatibacter actinomycetemcomitans
Actinobacillus suis H91-0380
Pasteurella dagmatis ATCC 43325
Desulfovibrio cuneatus
Desulfovibrio desulfuricans ATCC 27774
Serratia sp. Strain DD3
Serratia fonticola RB-25
Erwinia amylovora str CFBP1430
Acinetobacter baumannii WC-323
Providencia stuartii ATCC 25827
Photobacterium profundum
Psychromonas ossibalaenae
Psychromonas sp. CNPT3
Photobacterium sp. AK15
Photobacterium damselae
Photobacterium phosphoreum ANT220
Photobacterium sp. SKA34
Pasteurella multocida
Pasteurella pneumotropica
Mannheimia succiniciproducens
Actinobacillus suis H91-0380
Haemophilus parasuis ZJ0906
Actinobacillus minor NM305
Vibrio anguillarum ATCC 68554
Xenorhabdus cabanillasii JM26
Xenorhabdus szentirmaii DSM 16338
Xenorhabdus nematophila ATCC 19061
Xenorhabdus bovienii SS-2004
Vibrio fischeri ATCC 700601
Vibrio vulnificus
Vibrio parahaemolyticus BB22OP
Vibrio furnissii DSM 14383
Salinivibrio costicola
Vibrio metschnikovii CIP 69.14
Vibrio proteolyticus NBRC 13287
Vibrio coralliilyticus ATCC BAA-450
Vibrio shilonii AK1
Grimontia sp. AK16
Psychromonas arctica
Vibrio cyclitrophicus
Photobacterium profundum
Photobacterium sp. AK15
Vibrio anguillarum ATCC 68554
Vibrio cholerae serotype O1
Vibrio cholerae O1 str Inaba G4222
Vibrio furnissii DSM 14383
Vibrio metschnikovii CIP 69.14
Serratia fonticola AU-AP2C
Yersinia enterocolitica
Pectobacterium carotovorum
Dickeya dadantii
Photorhabdus luminescens
Xenorhabdus cabanillasii JM26
Providencia burhodogranariea DSM 19968
Providencia alcalifaciens
Providencia rettgeri Dmel1
Morganella morganii
Proteus mirabilis
Enterobacteriaceae bacterium LSJC7
Enterobacter cloacae subsp. cloacae ATCC 13047
Serratia odorifera DSM 4582
Escherichia hermannii NBRC 105704
Cronobacter pulveris
Edwardsiella tarda
Serratia marcescens WW4
Escherichia coli
Aeromonas hydrophila 4AK4
Aeromonas salmonicida
Pectobacterium carotovorum
Dickeya dadantii
Pantoea sp. YR343
Pantoea sp. Sc1
Enterobacter asburiae str LF7a
Klebsiella pneumoniae
Escherichia coli K12
Escherichia coli 178200
Escherichia coli TA007
Salmonella enterica
Salmonella enterica
Salmonella typhimurium
Citrobacter freundii CFNIH1
Legionella longbeachae serogroup 1
Chlamydia muridarum str MoPn
Chlamydia trachomatis str D/UW-3/Cx
Chlamydia psittaci 10.1398.11
Chlamydia pecorum DBDeUG
Chlamydia pneumoniae
Chlamydia pneumoniae str LPCoLN
Chlamydophila felis
Chlamydia psittaci
Chlamydophila caviae str GPIC
Chlamydia avium 10DC88
Bacteroides fragilis str S6L5
Vibrio cholerae serotype O1
Clostridium sp CAG.729
Clostridium sp CAG.306
Brachyspira sp CAG.484
uncultured bacterium
Candidatus Odyssella thessalonicensis
uncultured bacterium
uncultured bacterium
Bacteroides sp. 1.1.30
Bacteroides eggerthii
Pedosphaera parvula str Ellin514
Leptospira borgpetersenii serovar Hardjo-bovis
Leptospira wolffii serovar Khorat
Leptospira licerasiae serovar Varillal
Leptonema illini DSM 21528
Leptospira vanthielii serovar Holland
Leptospira biflexa serovar Patoc 1
Thermosinus carboxydivorans Nor1
Anaerobaculum mobile ATCC BAA-54
Clostridium sp. Ade.TY
Clostridium carboxidivorans P7
Clostridium saccharobutylicum DSM 13864
Clostridium bifermentans ATCC 19299
Clostridium sp. CAG.557
Clostridium botulinum str Eklund 17B
Clostridium botulinum str Okra
Clostridium botulinum D str 1873
Clostridium novyi str NT
Piscirickettsia salmonis LF-89
Fictibacillus gelatini
Laceyella sacchari
Mannheimia haemolytica USMARC 2286
Haemophilus parasuis 12939
Pasteurella pneumotropica
Actinobacillus suis H91-0380
Pasteurella bettyae CCUG 2042
Gemella morbillorum M424
Lautropia mirabilis ATCC 51599
Selenomonas sp. FOBRC9
Veillonella montpellierensis
Veillonella parvula ATCC 10790
Bacillus pumilus
Bacillus subtilis str 168
Aneurinibacillus aneurinilyticus ATCC 12856
Bacillus cereus subsp. cytotoxis str NVH 391-98
Desulfotomaculum hydrothermale Lam5
Desulfotomaculum reducens
Oceanobacillus picturae
Bacillus megaterium
Bacillus sp. J33
Bacillus coagulans
Bacillus macauensis ZFHKF-1
Paenibacillus elgii
Bacillus licheniformis
Brevibacillus borstelensis AK1
Brevibacillus brevis
Brochothrix thermosphacta DSM 20171
Lactobacillus coryniformis
Macrococcus caseolyticus
Staphylococcus sp. HGB0015
Staphylococcus pseudintermedius
Staphylococcus lugdunensis
Staphylococcus carnosus
Staphylococcus epidermidis ATCC 35984
Staphylococcus simiae CCM 7213
Staphylococcus aureus
Staphylococcus aureus C0673
Alistipes onderdonkii
Butyricimonas synergistica
Odoribacter splanchnicus CAG.14
Bacteroides sp. CAG.20
Pedobacter glucosidilyticus
Pedobacter arcticus
Pedobacter oryzae
Pontibacter actiniarum
Pedobacter saltans ATCC 51119
Sphingobacterium paucimobilis HER1398
Pedobacter heparinus ATCC 13125
Desulfovibrio acrylicus
Desulfovibrio magneticus ATCC 700980
Borrelia coriaceae Co53
Borrelia parkeri SLO
Borrelia hermsii YBT
Borrelia miyamotoi FR64b
Borrelia persica
Borrelia duttonii
Pseudoalteromonas atlantica
Glaciecola psychrophila
Aeromonas diversa
Aeromonas veronii
Salinivibrio costicola
Photobacterium phosphoreum ANT220
Psychromonas arctica
Psychromonas ossibalaenae
Photobacterium profundum
Photobacterium sp. AK15
Vibrio azureus
Vibrio sp. F6
Vibrio parahaemolyticus
Vibrio fischeri
Vibrio rumoiensis
Photobacterium angustum
Vibrio gazogenes
Vibrio metschnikovii CIP 69.14
Vibrio cholerae serotype O1
Vibrio cholerae
Vibrio anguillarum
Aliivibrio salmonicida
Moritella sp. PE36
Chromobacterium violaceum ATCC 12472
Mannheimia granulomatis
Haemophilus influenzae
Serratia sp.
Candidatus Regiella insecticola R5.15
Erwinia tracheiphila PSU-1
Shimwellia blattae ATCC 29907
Salmonella enterica subsp. enterica serovar Bareilly
Escherichia coli K12
Tatumella ptyseos ATCC 33301
Rahnella aquatilis ATCC 33071
Erwinia billingiae
Xenorhabdus szentirmaii DSM 16338
Mucispirillum schaedleri ASF457
Xenorhabdus bovienii
Photorhabdus luminescens subsp. laumondii strain TT01
Arsenophonus nasoniae
Anaerobiospirillum succiniciproducens
Morganella morganii subsp. morganii KT
Proteus mirabilis
Cobetia crustatorum
Pseudomonas psychrotolerans L19
Methylobacterium radiotolerans ATCC 27329
Pseudomonas sp. HPB0071
Pseudomonas aeruginosa PA96
Pseudomonas fulva
Pseudomonas syringae
Pseudomonas entomophila
Pseudomonas fluorescens NCIMB 11764
Pseudomonas sp. GM78
Pseudomonas fluorescens F113
Pseudomonas mandelii JR-1
0.2

Supplementary Figure 4: **Phylogenetic analysis of the UhpC gene under the CAT+GTR model.** This is the complete tree upon which Fig. 3(d) is based. Archaeplastida sequences are denoted with green squares, *Chlamydiae* with orange triangles, and other bacterial groups with black circles. Branch supports are Bayesian posterior probabilities, and branch lengths are proportional to the expected number of substitutions per site, as indicated by the scale bar.

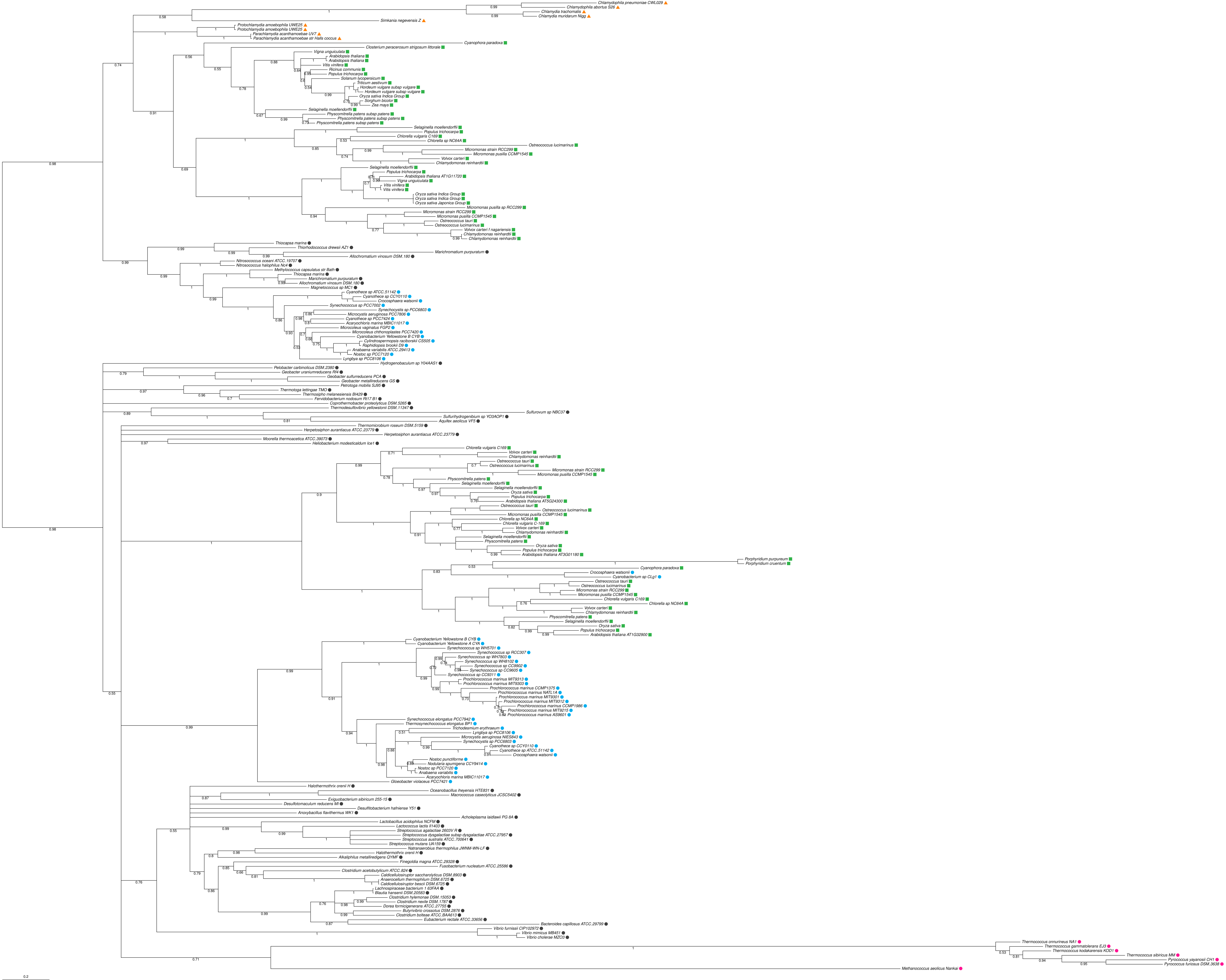

Chlamydophila pneumoniae CWL029
Chlamydophila abortus S26
Chlamydia trachomatis
Chlamydia muridarum Nigg
Simkania negevensis Z
Protochlamydia amoebophila UWE25
Protochlamydia amoebophila UWE25
Parachlamydia acanthamoebae UV7
Parachlamydia acanthamoebae str Halls coccus
Cyanophora paradoxa
Closterium peracerosum strigosum littorale
Vigna unguiculata
Arabidopsis thaliana
Arabidopsis thaliana
Vitis vinifera
Ricinus communis
Populus trichocarpa
Solanum lycopersicum
Triticum aestivum
Hordeum vulgare subsp vulgare
Hordeum vulgare subsp vulgare
Oryza sativa Indica Group
Sorghum bicolor
Zea mays
Selaginella moellendorffii
Physcomitrella patens subsp patens
Physcomitrella patens subsp patens
Physcomitrella patens subsp patens
Selaginella moellendorffii
Populus trichocarpa
Chlorella vulgaris C169
Chlorella sp NC64A
Ostreococcus lucimarinus
Micromonas strain RCC299
Micromonas pusilla CCMP1545
Volvox carteri
Chlamydomonas reinhardtii
Selaginella moellendorffii
Populus trichocarpa
Arabidopsis thaliana AT1G11720
Vigna unguiculata
Vitis vinifera
Vitis vinifera
Oryza sativa Indica Group
Oryza sativa Indica Group
Oryza sativa Japonica Group
Micromonas pusilla sp RCC299
Micromonas strain RCC299
Micromonas pusilla CCMP1545
Ostreococcus tauri
Ostreococcus lucimarinus
Volvox carteri f nagariensis
Chlamydomonas reinhardtii
Chlamydomonas reinhardtii
Thiocapsa marina
Thiorhodococcus drewsii AZ1
Marichromatium purpuratum
Allochromatium vinosum DSM.180
Nitrosococcus oceani ATCC.19707
Nitrosococcus halophilus Nc4
Methylococcus capsulatus str Bath
Thiocapsa marina
Marichromatium purpuratum
Allochromatium vinosum DSM.180
Magnetococcus sp MC1
Cyanothece sp ATCC.51142
Cyanothece sp CCY0110
Crocosphaera watsonii
Synechococcus sp PCC7002
Synechocystis sp PCC6803
Microcystis aeruginosa PCC7806
Cyanothece sp PCC7424
Acaryochloris marina MBIC11017
Microcoleus vaginatus FGP2
Microcoleus chthonoplastes PCC7420
Cyanobacterium Yellowstone B CYB
Cylindrospermopsis raciborskii CS505
Raphidiopsis brookii D9
Anabaena variabilis ATCC.29413
Nostoc sp PCC7120
Lyngbya sp PCC8106
Hydrogenobaculum sp Y04AAS1
Pelobacter carbinolicus DSM.2380
Geobacter uraniumreducens Rf4
Geobacter sulfurreducens PCA
Geobacter metallireducens GS
Petrotoga mobilis SJ95
Thermotoga lettingae TMO
Thermosipho melanesiensis BI429
Fervidobacterium nodosum Rt17 B1
Coprothermobacter proteolyticus DSM.5265
Thermodesulfovibrio yellowstonii DSM.11347
Sulfurovum sp NBC37
Sulfurihydrogenibium sp YO3AOP1
Aquifex aeolicus VF5
Thermomicrobium roseum DSM.5159
Herpetosiphon aurantiacus ATCC.23779
Herpetosiphon aurantiacus ATCC.23779
Moorella thermoacetica ATCC.39073
Heliobacterium modesticaldum Ice1
Chlorella vulgaris C169
Volvox carteri
Chlamydomonas reinhardtii
Ostreococcus tauri
Ostreococcus lucimarinus
Micromonas strain RCC299
Micromonas pusilla CCMP1545
Physcomitrella patens
Selaginella moellendorffii
Selaginella moellendorffii
Oryza sativa
Populus trichocarpa
Arabidopsis thaliana AT5G24300
Ostreococcus tauri
Ostreococcus lucimarinus
Micromonas pusilla CCMP1545
Chlorella sp NC64A
Chlorella vulgaris C-169
Volvox carteri
Chlamydomonas reinhardtii
Selaginella moellendorffii
Physcomitrella patens
Oryza sativa
Populus trichocarpa
Arabidopsis thaliana AT3G01180
Porphyridium purpureum
Porphyridium cruentum
Cyanophora paradoxa
Crocosphaera watsonii
Cyanobacterium sp CLg1
Ostreococcus tauri
Ostreococcus lucimarinus
Micromonas strain RCC299
Micromonas pusilla CCMP1545
Chlorella vulgaris C169
Chlorella sp NC64A
Volvox carteri
Chlamydomonas reinhardtii
Physcomitrella patens
Selaginella moellendorffii
Oryza sativa
Populus trichocarpa
Arabidopsis thaliana AT1G32900
Cyanobacterium Yellowstone B CYB
Cyanobacterium Yellowstone A CYA
Synechococcus sp WH5701
Synechococcus sp RCC307
Synechococcus sp WH7803
Synechococcus sp WH8102
Synechococcus sp CC9902
Synechococcus sp CC9605
Synechococcus sp CC9311
Prochlorococcus marinus MIT9313
Prochlorococcus marinus MIT9303
Prochlorococcus marinus CCMP1375
Prochlorococcus marinus NATL1A
Prochlorococcus marinus MIT9301
Prochlorococcus marinus MIT9312
Prochlorococcus marinus CCMP1986
Prochlorococcus marinus MIT9215
Prochlorococcus marinus AS9601
Synechococcus elongatus PCC7942
Thermosynechococcus elongatus BP1
Trichodesmium erythraeum
Lyngbya sp PCC8106
Microcystis aeruginosa NIES843
Synechocystis sp PCC6803
Cyanothece sp CCY0110
Cyanothece sp ATCC.51142
Crocosphaera watsonii
Nostoc punctiforme
Nodularia spumigena CCY9414
Nostoc sp PCC7120
Anabaena variabilis
Acaryochloris marina MBIC11017
Gloeobacter violaceus PCC7421
Halothermothrix orenii H
Oceanobacillus iheyensis HTE831
Macrococcus caseolyticus JCSC5402
Exiguobacterium sibiricum 255-15
Desulfotomaculum reducens MI
Desulfitobacterium hafniense Y51
Anoxybacillus flavithermus WK1
Acholeplasma laidlawii PG 8A
Lactobacillus acidophilus NCFM
Lactococcus lactis Il1403
Streptococcus agalactiae 2603V R
Streptococcus dysgalactiae subsp dysgalactiae ATCC.27957
Streptococcus australis ATCC.700641
Streptococcus mutans UA159
Natranaerobius thermophilus JWNM-WN-LF
Halothermothrix orenii H
Alkaliphilus metalliredigens QYMF
Finegoldia magna ATCC.29328
Fusobacterium nucleatum ATCC.25586
Clostridium acetobutylicum ATCC.824
Caldicellulosiruptor saccharolyticus DSM.8903
Anaerocellum thermophilum DSM.6725
Caldicellulosiruptor bescii DSM.6725
Lachnospiraceae bacterium 1 63FAA
Blautia hansenii DSM.20583
Clostridium hylemonae DSM.15053
Clostridium nexile DSM.1787
Dorea formicigenerans ATCC.27755
Butyrivibrio crossotus DSM.2876
Clostridium bolteae ATCC.BAA613
Eubacterium rectale ATCC.33656
Bacteroides capillosus ATCC.29799
Vibrio furnissii CIP102972
Vibrio mimicus MB451
Vibrio cholerae MZO3
Thermococcus onnurineus NA1
Thermococcus gammatolerans EJ3
Thermococcus kodakarensis KOD1
Thermococcus sibiricus MM
Pyrococcus yayanosii CH1
Pyrococcus furiosus DSM.3638
Methanococcus aeolicus Nankai
0.2

Supplementary Figure 5: **Phylogenetic analysis of the GlgA gene under the CAT+GTR model.** This is the complete tree upon which Fig. 4(a) is based. Archaeplastida sequences are denoted with green squares, *Chlamydiae* with orange triangles, *Cyanobacteria* with cyan circles, other bacterial groups with black circles, and Archaea with pink circles. Branch supports are Bayesian posterior probabilities, and branch lengths are proportional to the expected number of substitutions per site, as indicated by the scale bar.

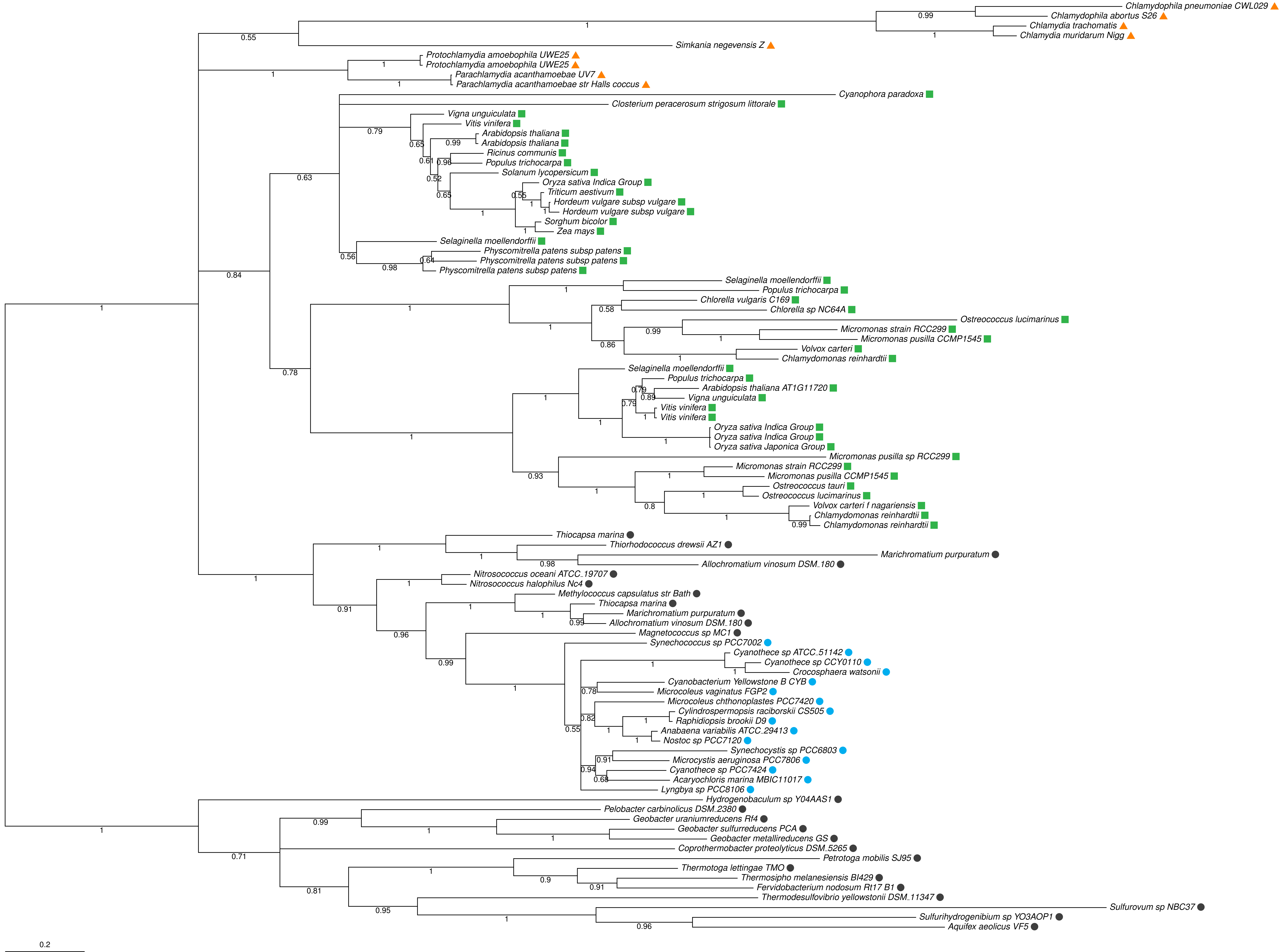
Chlamydophila pneumoniae CWL029
Chlamydophila abortus S26
Chlamydia trachomatis
Chlamydia muridarum Nigg
Simkania negevensis Z
Protochlamydia amoebophila UWE25
Protochlamydia amoebophila UWE25
Parachlamydia acanthamoebae UV7
Parachlamydia acanthamoebae str Halls coccus
Cyanophora paradoxa
Closterium peracerosum strigosum littorale
Vigna unguiculata
Vitis vinifera
Arabidopsis thaliana
Arabidopsis thaliana
Ricinus communis
Populus trichocarpa
Solanum lycopersicum
Oryza sativa Indica Group
Triticum aestivum
Hordeum vulgare subsp vulgare
Hordeum vulgare subsp vulgare
Sorghum bicolor
Zea mays
Selaginella moellendorffii
Physcomitrella patens subsp patens
Physcomitrella patens subsp patens
Physcomitrella patens subsp patens
Selaginella moellendorffii
Populus trichocarpa
Chlorella vulgaris C169
Chlorella sp NC64A
Ostreococcus lucimarinus
Micromonas strain RCC299
Micromonas pusilla CCMP1545
Volvox carteri
Chlamydomonas reinhardtii
Selaginella moellendorffii
Populus trichocarpa
Arabidopsis thaliana AT1G11720
Vigna unguiculata
Vitis vinifera
Vitis vinifera
Oryza sativa Indica Group
Oryza sativa Indica Group
Oryza sativa Japonica Group
Micromonas pusilla sp RCC299
Micromonas strain RCC299
Micromonas pusilla CCMP1545
Ostreococcus tauri
Ostreococcus lucimarinus
Volvox carteri f nagariensis
Chlamydomonas reinhardtii
Chlamydomonas reinhardtii
Thiocapsa marina
Thiorhodococcus drewsii AZ1
Marichromatium purpuratum
Allochromatium vinosum DSM_180
Nitrosococcus oceani ATCC_19707
Nitrosococcus halophilus Nc4
Methylococcus capsulatus str Bath
Thiocapsa marina
Marichromatium purpuratum
Allochromatium vinosum DSM_180
Magnetococcus sp MC1
Synechococcus sp PCC7002
Cyanothece sp ATCC_51142
Cyanothece sp CCY0110
Crocosphaera watsonii
Cyanobacterium Yellowstone B CYB
Microcoleus vaginatus FGP2
Microcoleus chthonoplastes PCC7420
Cylindrospermopsis raciborskii CS505
Raphidiopsis brookii D9
Anabaena variabilis ATCC_29413
Nostoc sp PCC7120
Synechocystis sp PCC6803
Microcystis aeruginosa PCC7806
Cyanothece sp PCC7424
Acaryochloris marina MBIC11017
Lyngbya sp PCC8106
Hydrogenobaculum sp Y04AAS1
Pelobacter carbinolicus DSM_2380
Geobacter uraniumreducens Rf4
Geobacter sulfurreducens PCA
Geobacter metallireducens GS
Coprothermobacter proteolyticus DSM_5265
Petrotoga mobilis SJ95
Thermotoga lettingae TMO
Thermosipho melanesiensis BI429
Fervidobacterium nodosum Rt17 B1
Thermodesulfovibrio yellowstonii DSM_11347
Sulfurovum sp NBC37
Sulfurihydrogenibium sp YO3AOP1
Aquifex aeolicus VF5
0.2

Supplementary Figure 6: **Phylogenetic analysis of the GlgA gene with only the closest outgroup clade under the CAT+GTR model.** This is the complete tree upon which Fig. 4(b) is based. Archaeplastida sequences are denoted with green squares, other eukaryotes with purple circles, *Chlamydiae* with orange triangles, *Cyanobacteria* with cyan circles, and other bacterial groups with black circles. Branch supports are Bayesian posterior probabilities, and branch lengths are proportional to the expected number of substitutions per site, as indicated by the scale bar.

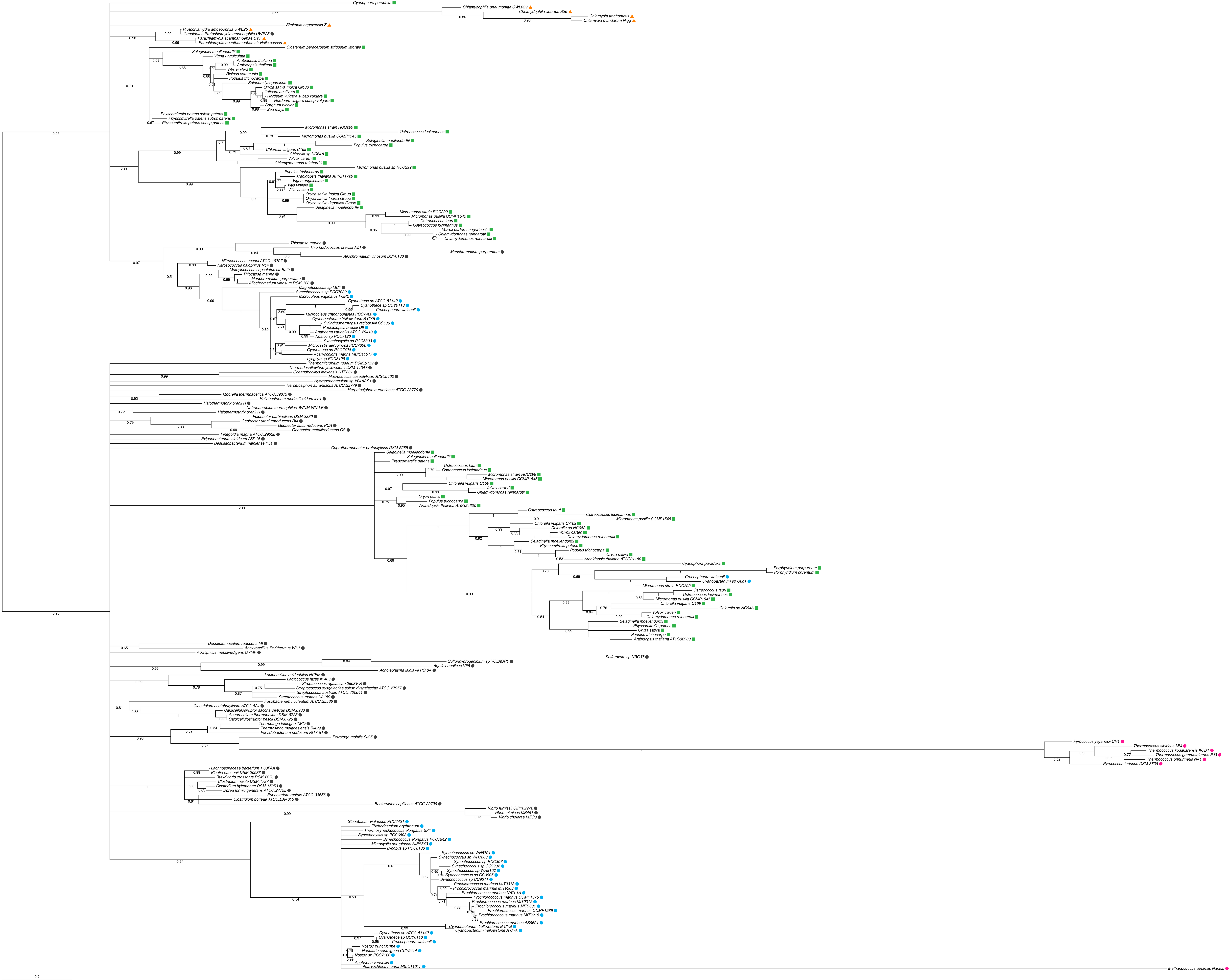
Cyanophora paradoxa
Chlamydophila pneumoniae CWL029
Chlamydophila abortus S26
Chlamydia trachomatis
Chlamydia muridarum Nigg
Simkania negevensis Z
Protochlamydia amoebophila UWE25
Candidatus Protochlamydia amoebophila UWE25
Parachlamydia acanthamoebae UV7
Parachlamydia acanthamoebae str Halls coccus
Closterium peracerosum strigosum littorale
Selaginella moellendorffii
Vigna unguiculata
Arabidopsis thaliana
Arabidopsis thaliana
Vitis vinifera
Ricinus communis
Populus trichocarpa
Solanum lycopersicum
Oryza sativa Indica Group
Triticum aestivum
Hordeum vulgare subsp vulgare
Hordeum vulgare subsp vulgare
Sorghum bicolor
Zea mays
Physcomitrella patens subsp patens
Physcomitrella patens subsp patens
Physcomitrella patens subsp patens
Micromonas strain RCC299
Ostreococcus lucimarinus
Micromonas pusilla CCMP1545
Selaginella moellendorffii
Populus trichocarpa
Chlorella vulgaris C169
Chlorella sp NC64A
Volvox carteri
Chlamydomonas reinhardtii
Micromonas pusilla sp RCC299
Populus trichocarpa
Arabidopsis thaliana AT1G11720
Vigna unguiculata
Vitis vinifera
Vitis vinifera
Oryza sativa Indica Group
Oryza sativa Indica Group
Oryza sativa Japonica Group
Selaginella moellendorffii
Micromonas strain RCC299
Micromonas pusilla CCMP1545
Ostreococcus tauri
Ostreococcus lucimarinus
Volvox carteri f nagariensis
Chlamydomonas reinhardtii
Chlamydomonas reinhardtii
Thiocapsa marina
Thiorhodococcus drewsii AZ1
Marichromatium purpuratum
Allochromatium vinosum DSM.180
Nitrosococcus oceani ATCC.19707
Nitrosococcus halophilus Nc4
Methylococcus capsulatus str Bath
Thiocapsa marina
Marichromatium purpuratum
Allochromatium vinosum DSM.180
Magnetococcus sp MC1
Synechococcus sp PCC7002
Microcoleus vaginatus FGP2
Cyanothece sp ATCC.51142
Cyanothece sp CCY0110
Crocosphaera watsonii
Microcoleus chthonoplastes PCC7420
Cyanobacterium Yellowstone B CYB
Cylindrospermopsis raciborskii CS505
Raphidiopsis brookii D9
Anabaena variabilis ATCC.29413
Nostoc sp PCC7120
Synechocystis sp PCC6803
Microcystis aeruginosa PCC7806
Cyanothece sp PCC7424
Acaryochloris marina MBIC11017
Lyngbya sp PCC8106
Thermomicrobium roseum DSM.5159
Thermodesulfovibrio yellowstonii DSM.11347
Oceanobacillus iheyensis HTE831
Macrococcus caseolyticus JCSC5402
Hydrogenobaculum sp Y04AAS1
Herpetosiphon aurantiacus ATCC.23779
Herpetosiphon aurantiacus ATCC.23779
Moorella thermoacetica ATCC.39073
Heliobacterium modesticaldum Ice1
Halothermothrix orenii H
Natranaerobius thermophilus JWNM-WN-LF
Halothermothrix orenii H
Pelobacter carbinolicus DSM.2380
Geobacter uraniumreducens Rf4
Geobacter sulfurreducens PCA
Geobacter metallireducens GS
Finegoldia magna ATCC.29328
Exiguobacterium sibiricum 255-15
Desulfitobacterium hafniense Y51
Coprothermobacter proteolyticus DSM.5265
Selaginella moellendorffii
Selaginella moellendorffii
Physcomitrella patens
Ostreococcus tauri
Ostreococcus lucimarinus
Micromonas strain RCC299
Micromonas pusilla CCMP1545
Chlorella vulgaris C169
Volvox carteri
Chlamydomonas reinhardtii
Oryza sativa
Populus trichocarpa
Arabidopsis thaliana AT5G24300
Ostreococcus tauri
Ostreococcus lucimarinus
Micromonas pusilla CCMP1545
Chlorella vulgaris C-169
Chlorella sp NC64A
Volvox carteri
Chlamydomonas reinhardtii
Selaginella moellendorffii
Physcomitrella patens
Populus trichocarpa
Oryza sativa
Arabidopsis thaliana AT3G01180
Cyanophora paradoxa
Porphyridium purpureum
Porphyridium cruentum
Crocosphaera watsonii
Cyanobacterium sp CLg1
Micromonas strain RCC299
Ostreococcus tauri
Ostreococcus lucimarinus
Micromonas pusilla CCMP1545
Chlorella vulgaris C169
Chlorella sp NC64A
Volvox carteri
Chlamydomonas reinhardtii
Selaginella moellendorffii
Physcomitrella patens
Oryza sativa
Populus trichocarpa
Arabidopsis thaliana AT1G32900
Desulfotomaculum reducens MI
Anoxybacillus flavithermus WK1
Alkaliphilus metalliredigens QYMF
Sulfurovum sp NBC37
Sulfurihydrogenibium sp YO3AOP1
Aquifex aeolicus VF5
Acholeplasma laidlawii PG 8A
Lactobacillus acidophilus NCFM
Lactococcus lactis Il1403
Streptococcus agalactiae 2603V R
Streptococcus dysgalactiae subsp dysgalactiae ATCC.27957
Streptococcus australis ATCC.700641
Streptococcus mutans UA159
Fusobacterium nucleatum ATCC.25586
Clostridium acetobutylicum ATCC.824
Caldicellulosiruptor saccharolyticus DSM.8903
Anaerocellum thermophilum DSM.6725
Caldicellulosiruptor bescii DSM.6725
Thermotoga lettingae TMO
Thermosipho melanesiensis BI429
Fervidobacterium nodosum Rt17 B1
Petrotoga mobilis SJ95
Pyrococcus yayanosii CH1
Thermococcus sibiricus MM
Thermococcus kodakarensis KOD1
Thermococcus gammatolerans EJ3
Thermococcus onnurineus NA1
Pyrococcus furiosus DSM.3638
Lachnospiraceae bacterium 1 63FAA
Blautia hansenii DSM.20583
Butyrivibrio crossotus DSM.2876
Clostridium nexile DSM.1787
Clostridium hylemonae DSM.15053
Dorea formicigenerans ATCC.27755
Eubacterium rectale ATCC.33656
Clostridium bolteae ATCC.BAA613
Bacteroides capillosus ATCC.29799
Vibrio furnissii CIP102972
Vibrio mimicus MB451
Vibrio cholerae MZO3
Gloeobacter violaceus PCC7421
Trichodesmium erythraeum
Thermosynechococcus elongatus BP1
Synechocystis sp PCC6803
Synechococcus elongatus PCC7942
Microcystis aeruginosa NIES843
Lyngbya sp PCC8106
Synechococcus sp WH5701
Synechococcus sp WH7803
Synechococcus sp RCC307
Synechococcus sp CC9902
Synechococcus sp WH8102
Synechococcus sp CC9605
Synechococcus sp CC9311
Prochlorococcus marinus MIT9313
Prochlorococcus marinus MIT9303
Prochlorococcus marinus NATL1A
Prochlorococcus marinus CCMP1375
Prochlorococcus marinus MIT9312
Prochlorococcus marinus MIT9301
Prochlorococcus marinus CCMP1986
Prochlorococcus marinus MIT9215
Prochlorococcus marinus AS9601
Cyanobacterium Yellowstone B CYB
Cyanobacterium Yellowstone A CYA
Cyanothece sp ATCC.51142
Cyanothece sp CCY0110
Crocosphaera watsonii
Nostoc punctiforme
Nodularia spumigena CCY9414
Nostoc sp PCC7120
Anabaena variabilis
Acaryochloris marina MBIC11017
Methanococcus aeolicus Nankai
0.2

Supplementary Figure 7: **Phylogenetic analysis of the Dayhoff-recoded GlgA alignment under the CAT+GTR model.** This is the complete tree upon which Fig. 4(c) is based. Archaeplastida sequences are denoted with green squares, *Chlamydiae* with orange triangles, *Cyanobacteria* with cyan circles, other bacterial groups with black circles, and Archaea with pink circles. Branch supports are Bayesian posterior probabilities, and branch lengths are proportional to the expected number of substitutions per site, as indicated by the scale bar.

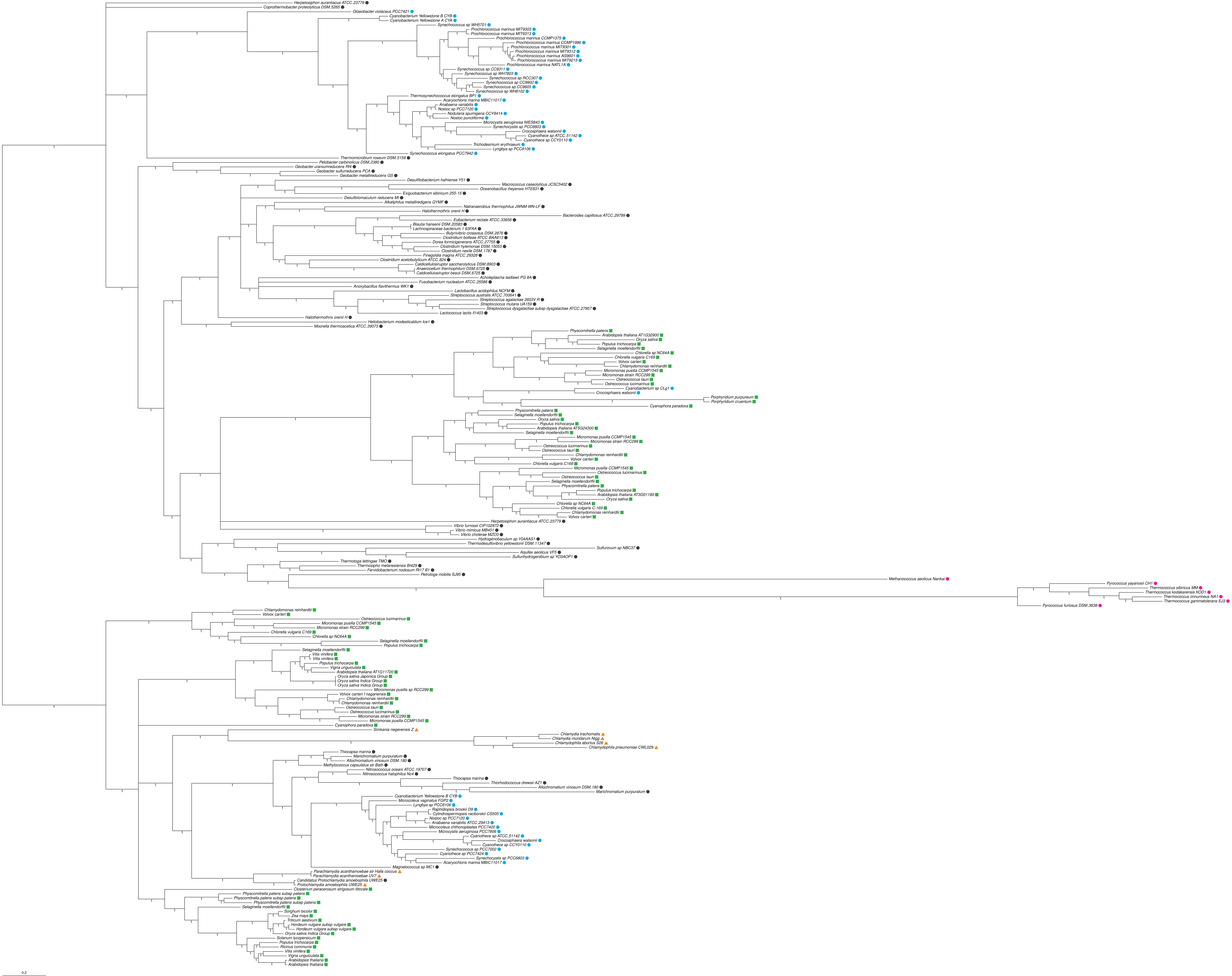

Herpetosiphon aurantiacus ATCC.23779
Coprothermobacter proteolyticus DSM.5265
Gloeobacter violaceus PCC7421
Cyanobacterium Yellowstone B CYB
Cyanobacterium Yellowstone A CYA
Synechococcus sp WH5701
Prochlorococcus marinus MIT9303
Prochlorococcus marinus MIT9313
Prochlorococcus marinus CCMP1375
Prochlorococcus marinus CCMP1986
Prochlorococcus marinus MIT9301
Prochlorococcus marinus MIT9312
Prochlorococcus marinus AS9601
Prochlorococcus marinus MIT9215
Prochlorococcus marinus NATL1A
Synechococcus sp CC9311
Synechococcus sp WH7803
Synechococcus sp RCC307
Synechococcus sp CC9902
Synechococcus sp CC9605
Synechococcus sp WH8102
Thermosynechococcus elongatus BP1
Acaryochloris marina MBIC11017
Anabaena variabilis
Nostoc sp PCC7120
Nodularia spumigena CCY9414
Nostoc punctiforme
Microcystis aeruginosa NIES843
Synechocystis sp PCC6803
Crocosphaera watsonii
Cyanothece sp ATCC.51142
Cyanothece sp CCY0110
Trichodesmium erythraeum
Lyngbya sp PCC8106
Synechococcus elongatus PCC7942
Thermomicrobium roseum DSM.5159
Pelobacter carbinolicus DSM.2380
Geobacter uraniumreducens Rf4
Geobacter sulfurreducens PCA
Geobacter metallireducens GS
Desulfitobacterium hafniense Y51
Macrococcus caseolyticus JCSC5402
Oceanobacillus iheyensis HTE831
Exiguobacterium sibiricum 255-15
Desulfotomaculum reducens MI
Alkaliphilus metalliredigens QYMF
Natranaerobius thermophilus JWNM-WN-LF
Halothermothrix orenii H
Bacteroides capillosus ATCC.29799
Eubacterium rectale ATCC.33656
Blautia hansenii DSM.20583
Lachnospiraceae bacterium 1 63FAA
Butyrivibrio crossotus DSM.2876
Clostridium bolteae ATCC.BAA613
Dorea formicigenerans ATCC.27755
Clostridium hylemonae DSM.15053
Clostridium nexile DSM.1787
Finegoldia magna ATCC.29328
Clostridium acetobutylicum ATCC.824
Caldicellulosiruptor saccharolyticus DSM.8903
Anaerocellum thermophilum DSM.6725
Caldicellulosiruptor bescii DSM.6725
Acholeplasma laidlawii PG 8A
Fusobacterium nucleatum ATCC.25586
Anoxybacillus flavithermus WK1
Lactobacillus acidophilus NCFM
Streptococcus australis ATCC.700641
Streptococcus agalactiae 2603V R
Streptococcus mutans UA159
Streptococcus dysgalactiae subsp dysgalactiae ATCC.27957
Lactococcus lactis Il1403
Halothermothrix orenii H
Heliobacterium modesticaldum Ice1
Moorella thermoacetica ATCC.39073
Physcomitrella patens
Arabidopsis thaliana AT1G32900
Oryza sativa
Populus trichocarpa
Selaginella moellendorffii
Chlorella sp NC64A
Chlorella vulgaris C169
Volvox carteri
Chlamydomonas reinhardtii
Micromonas pusilla CCMP1545
Micromonas strain RCC299
Ostreococcus tauri
Ostreococcus lucimarinus
Cyanobacterium sp CLg1
Crocosphaera watsonii
Porphyridium purpureum
Porphyridium cruentum
Cyanophora paradoxa
Physcomitrella patens
Selaginella moellendorffii
Oryza sativa
Populus trichocarpa
Arabidopsis thaliana AT5G24300
Selaginella moellendorffii
Micromonas pusilla CCMP1545
Micromonas strain RCC299
Ostreococcus lucimarinus
Ostreococcus tauri
Chlamydomonas reinhardtii
Volvox carteri
Chlorella vulgaris C169
Micromonas pusilla CCMP1545
Ostreococcus lucimarinus
Ostreococcus tauri
Selaginella moellendorffii
Physcomitrella patens
Populus trichocarpa
Arabidopsis thaliana AT3G01180
Oryza sativa
Chlorella sp NC64A
Chlorella vulgaris C-169
Chlamydomonas reinhardtii
Volvox carteri
Herpetosiphon aurantiacus ATCC.23779
Vibrio furnissii CIP102972
Vibrio mimicus MB451
Vibrio cholerae MZO3
Hydrogenobaculum sp Y04AAS1
Thermodesulfovibrio yellowstonii DSM.11347
Sulfurovum sp NBC37
Aquifex aeolicus VF5
Sulfurihydrogenibium sp YO3AOP1
Thermotoga lettingae TMO
Thermosipho melanesiensis BI429
Fervidobacterium nodosum Rt17 B1
Petrotoga mobilis SJ95
Methanococcus aeolicus Nankai
Pyrococcus yayanosii CH1
Thermococcus sibiricus MM
Thermococcus kodakarensis KOD1
Thermococcus onnurineus NA1
Thermococcus gammatolerans EJ3
Pyrococcus furiosus DSM.3638
Chlamydomonas reinhardtii
Volvox carteri
Ostreococcus lucimarinus
Micromonas pusilla CCMP1545
Micromonas strain RCC299
Chlorella vulgaris C169
Chlorella sp NC64A
Selaginella moellendorffii
Populus trichocarpa
Selaginella moellendorffii
Vitis vinifera
Vitis vinifera
Populus trichocarpa
Vigna unguiculata
Arabidopsis thaliana AT1G11720
Oryza sativa Japonica Group
Oryza sativa Indica Group
Oryza sativa Indica Group
Micromonas pusilla sp RCC299
Volvox carteri f nagariensis
Chlamydomonas reinhardtii
Chlamydomonas reinhardtii
Ostreococcus tauri
Ostreococcus lucimarinus
Micromonas strain RCC299
Micromonas pusilla CCMP1545
Cyanophora paradoxa
Simkania negevensis Z
Chlamydia trachomatis
Chlamydia muridarum Nigg
Chlamydophila abortus S26
Chlamydophila pneumoniae CWL029
Thiocapsa marina
Marichromatium purpuratum
Allochromatium vinosum DSM.180
Methylococcus capsulatus str Bath
Nitrosococcus oceani ATCC.19707
Nitrosococcus halophilus Nc4
Thiocapsa marina
Thiorhodococcus drewsii AZ1
Allochromatium vinosum DSM.180
Marichromatium purpuratum
Cyanobacterium Yellowstone B CYB
Microcoleus vaginatus FGP2
Lyngbya sp PCC8106
Raphidiopsis brookii D9
Cylindrospermopsis raciborskii CS505
Nostoc sp PCC7120
Anabaena variabilis ATCC.29413
Microcoleus chthonoplastes PCC7420
Microcystis aeruginosa PCC7806
Cyanothece sp ATCC.51142
Crocosphaera watsonii
Cyanothece sp CCY0110
Synechococcus sp PCC7002
Cyanothece sp PCC7424
Synechocystis sp PCC6803
Acaryochloris marina MBIC11017
Magnetococcus sp MC1
Parachlamydia acanthamoebae str Halls coccus
Parachlamydia acanthamoebae UV7
Candidatus Protochlamydia amoebophila UWE25
Protochlamydia amoebophila UWE25
Closterium peracerosum strigosum littorale
Physcomitrella patens subsp patens
Physcomitrella patens subsp patens
Physcomitrella patens subsp patens
Selaginella moellendorffii
Sorghum bicolor
Zea mays
Triticum aestivum
Hordeum vulgare subsp vulgare
Hordeum vulgare subsp vulgare
Oryza sativa Indica Group
Solanum lycopersicum
Populus trichocarpa
Ricinus communis
Vitis vinifera
Vigna unguiculata
Arabidopsis thaliana
Arabidopsis thaliana
0.2

Supplementary Figure 8: **Phylogenetic analysis of the GlgA gene under the nonstationary CAT+BP model.** This is the complete tree upon which Fig. 4(d) is based. Archaeplastida sequences are denoted with green squares, other eukaryotes with purple circles, *Chlamydiae* with orange triangles, *Cyanobacteria* with cyan circles, other bacterial groups with black circles, and Archaea with pink circles. Branch supports are Bayesian posterior probabilities, and branch lengths are proportional to the expected number of substitutions per site, as indicated by the scale bar.

Supplementary Table 1: Posterior predictive simulations of compositional heterogeneity for the single gene alignments analyzed in this study.

	Across-site composition			Across-branch composition		
	Observed	Predicted	*P*-value	Observed	Predicted	*P*-value
**GlgC**						
LG	7.78	9.71 +/- 0.1	**0**	0.0016	0.0014	0.069
CAT+GTR	7.78	7.86 +/- 0.19	0.33	0.004	0.0047 +/- 0.0009	0.63
**GlgP**						
LG	8.51	11.61 +/- 0.09	**0**	0.008	0.0026	**0**
CAT+GTR	8.51	8.91 +/- 0.09	**0**	0.008	0.0049 +/- 0.00057	**0**
**GlgX**						
LG	9.43	11.17 +/- 0.09	**0**	0.015	0.004	**0**
CAT+GTR	9.43	9.54 +/- 0.16697	0.24	0.015	0.0059 +/- 0.001	**0**
**UhpC**						
LG	8.465	9.84 +/- 0.14	**0**	0.009	0.0069 +/- 0.0007	**0.008**
CAT+GTR	8.465	8.41 +/- 0.09	0.71	0.009	0.0068 +/- 0.0008	**0.008**
**GlgA**						
LG	10.97	13.64 +/- 0.11	**0**	0.016	0.0083	**0.002**
CAT+GTR	10.97	11.49 +/- 0.16	**0**	0.016	0.009	**0.017**
CAT+GTR (closest outgroup)	8.253	8.414 +/- 0.2	0.21	0.017	0.008 +/- 0.001	**0**
CAT+GTR +Dayhoff	4.409	4.408 +/- 0.04	0.49	0.012	0.008	0.1
